# Traditional and confocal descriptions of a new genus and two new species of deep water Cerviniinae Sars, 1903 from the Southern Atlantic and the Norwegian Sea: with a discussion on the use of digital media in taxonomy (Copepoda, Harpacticoida, Aegisthidae)

**DOI:** 10.3897/zookeys.766.23899

**Published:** 2018-06-13

**Authors:** Paulo H. C. Corgosinho, Terue C. Kihara, Nikolaos V. Schizas, Alexandra Ostmann, Pedro Martínez Arbizu, Viatcheslav N. Ivanenko

**Affiliations:** 1 Department of General Biology, State University of Montes Claros (UNIMONTES), Campus Universitário Professor Darcy Ribeiro, 39401-089 Montes Claros (MG), Brazil; 2 Senckenberg am Meer, Department of German Center for Marine Biodiversity Research, Südstrand 44, 26382 Wilhelmshaven, Germany; 3 Department of Marine Sciences, University of Puerto Rico at Mayagüez, Call Box 9000, Mayagüez, PR 00681, USA; 4 Department of Invertebrate Zoology, Biological Faculty, Lomonosov Moscow State University, 119899 Moscow, Russia

**Keywords:** Arctic biodiversity, *Cerviniella*, deep-sea biodiversity, digital taxonomy, meiofauna, *Paracerviniella*, Tropical Atlantic biodiversity

## Abstract

Aegisthidae is one of the most abundant and diverse families of harpacticoid copepods living in deep-sea benthos, and the phylogenetic relationships within the family are in state of flux. Females of two new deep-water species of harpacticoid copepods belonging to the *Hase* **gen. n.** (Aegisthidae: Cerviniinae) are described. The first taxonomic description of marine copepod species based on the combined use of interference and confocal microscopy for the study of the habitus and dissected appendages is presented here. CLSM (Confocal Laser Scanning Microscopy) is a non-destructive method, comparable in quality to SEM (scanning electron microscopy) at the same magnifications. To observe and reconstruct in detail the habitus and dissected appendages, whole specimens and dissected parts were stained with Congo Red, mounted on slides with glycerine for CLSM and scanned under three visible-light lasers. *Hase lagomorphicus* **gen. et sp. n.** and *Hase talpamorphicus* **gen. et sp. n.** were collected from the sediments of the Southern Atlantic and the Norwegian Sea, from 2270 m and 5468 m depths, respectively. *Hase* **gen. n.** is included within Cerviniinae based on the caudal rami which are relatively divergent. *Hase* **gen. n.** is the sister taxon of *Cerviniella* based on the following synapomorphies: sturdy body, exopodites 1–3 of pereopods 1–3 heavily built, transformed into digging limbs, with strong outer and distal spines/setae, two-segmented endopod on the pereopods 2 and 3, and a reduced pereopod 5. Compared to *Cerviniella, Hase* **gen. n.** exhibits a more developed armature on the pereopod 1, which has outer and distal elements transformed into strong and long spines vs. stiff setae on *Cerviniella.Hase* **gen. n.** has one or two strong and long spines on the inner margin of the exopodite 3 of pereopod 4 and pereopod 5 is fused to the somite, ornamented with three distal setae. The telson of *Hase* **gen. n.** is subquadratic, and the furca is among the shortest yet described for Aegisthidae. The new species differ in a number of diagnostic characters, three of which are: a) the somite bearing pereopods 3 and 4 with latero-distal spiniform processes in *H. talpamorphicus* **gen. et sp. n.** but smooth in *H. lagomorphicus* **gen. et sp. n.**, b) antenna is armed with three stout spines on the lateral inner margin of the exopod in *H. talpamorphicus* **gen. et sp. n.** and two proximal setae in *H. lagomorphicus* **gen. et sp. n.**, and c) pereopod 4 exopodite 3 has two long and strong spines on the inner margin in *H. lagomorphicus* **gen. et sp. n.** and one spine in *H. talpamorphicus* **gen. et sp. n.** The high quality of CLSM images should foster discussion about the use of high quality digital images as type or as part of the type series in zoological studies, especially when studying rare and small macrofaunal and meiofaunal taxa.

## Introduction

Aegisthidae Giesbrecht, 1893 is one of the most abundant and diverse families of harpacticoid copepods living in deep-sea plankton and benthos (George et al. 2014). They are found in holoplankton, hyperbenthos, as well as hydrothermal vents and cold seeps (Giesbrecht 1891, Conroy-Dalton and Huys 1999, Lee and Huys 2000). According to Seifried and Schminke (2003) the Aegisthidae comprises three subfamilies: Aegisthinae with the genera *Aegisthus* Giesbrecht, 1891, *Andromastax* Conroy-Dalton & Huys, 1999, *Jamstecia* Lee & Huys, 2000, *Nudivorax* Lee & Huys, 2000, *Scabrantenna* Lee & Huys, 2000; Cerviniinae with the genera *Brodskaya* Huys, Møbjerg & Kristensen, 1997, *Cervinia* Norman, 1878, *Cerviniella* Smirnov, 1946, *Eucanuella* T. Scott, 1900, *Expansicervinia* Montagna, 1981, *Neocervinia* Huys, Møbjerg & Kristensen, 1997, *Paracerviniella* Brodskaya, 1963, *Pseudocervinia* Brodskaya, 1963; and Cerviniopseinae with the genera *Cerviniopsis* Sars, 1909, *Hemicervinia* Lang, 1935, *Herdmaniopsis* Brodskaya, 1963, *Pontostratiotes* Brady, 1883, *Stratiopontotes* Soyer, 1970, *Tonpostratiotes* Itô, 1982.

The phylogenetic relationships within the family Aegisthidae are in state of flux. According to Walter and Boxshall (2018), the family comprises 102 species in 18 genera and the four subfamilies Aegisthinae Giesbrecht, 1893, Cerviniinae Sars M., 1903, Cerviniopseinae Brotskaya, 1963, and Pontostratiotinae Scott, A., 1909. Seifried and Schminke (2003) suggested that the systematics of the group remains problematic, as species of Aegisthinae (formerly Aegisthidae) represent derived Cerviniopseinae. However, they decided to maintain the family division on Aegisthinae, Cerviniinae, and Cerviniopseinae until a more careful phylogenetic analysis is performed (Seifried and Schminke 2003, Kihara and Martínez Arbizu 2012). More recently, Huys (2009) reinstated the subfamily Pontostrationinae over Cerviniopseinae.

The paper describes two new species of copepod crustaceans designated to a new genus of the subfamily Cerviniinae (Harpacticoida: Aegisthidae) found in the deep waters of Southern Atlantic and Norwegian Sea. This is the first formal description of a marine copepod species based on combined use of interference and confocal microscopy in study of dissected appendages and the genital field. The methods for the acquisition of 3D rendered images are described by Corgosinho et al. (2017) and Kamanli et al. (2017). One of the most important advantages of using Confocal Laser Scanning Microscopy (CLSM) over Scanning Electron Microscopy (SEM) is that CLSM is a nondestructive imaging technique for the study of whole microscopic animals or small parts of them, such as millimetre or micrometre-long hydrated structures (see extensive discussion by Kamanli et al. 2017). In addition, the use of CLSM improves the presentation of appendages and structures in their natural 3-dimentional state, a property not easily transferable by the 2-dimentional inked drawings. High quality CLSM photos could substitute the need of taxonomists to acquire type specimens from Museums for comparisons, therefore lessening the burden of understaffed museums but also decrease the likelihood of a lost or damaged type material through the transfer of the specimens back and forth to the Museum. High quality CLSM photos depict structures as they appear in reality and potentially remove any shortcomings that an inexperienced taxonomist may have and will increase the quality of publications. The new species were sampled in the frame of the Census of Marine Life subproject CeDAMar (Census of the Diversity of Marine Abyssal Life, 2000–2010) and the IceAGE (Icelandic marine Animals: Genetics and Ecology, since 2011) project. The CeDAMar was a ten-year multinational project (from 2000 to 2010) devoted to map the world biodiversity in the abyssal plains between 4,000 to 5,000 meters deep. The aim of the IceAGE project was to combine classical taxonomic methods with modern biodiversity research, in particular phylogeography and ecological modelling in the climatically sensitive region around Iceland.

## Material and methods

**Figure 1.**
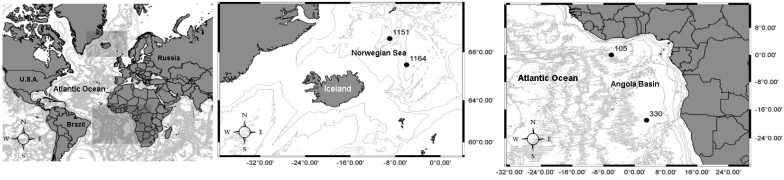
Sampling locations around Iceland (middle) and South Atlantic Ocean (right).

The copepods were extracted from sediment samples of three scientific cruises of the Research vessel (RV) “Meteor”. Sediment samples (5127–5455 m depth) were collected by a multi corer (MUC) during the DIVA-1 expedition of the RV “Meteor” (Cruise No. M48/1) to southeast Atlantic Ocean in July–August 2000. During DIVA-2 Expedition of the RV “Meteor” (Cruise No. M63/2), samples were taken by a MUC in the equatorial east Atlantic at depths >5000 m. Additional samples were collected by a box corer (BC) during the Overflow, Circulation and Biodiversity Expedition of the RV “Meteor” (Cruise No. M85/3) 307–2749 m deep (Fig. 1, Table 1), in the northernmost North Atlantic and the Arctic Ocean. Temperature and salinity were obtained by a CTD probe coupled to the MUC and BC.

**Table 1.** Sampling stations of the “Meteor” cruises. Abbreviations: BC – Box Corer, MUC – Multi Corer.

Species	Cruise	Station	Date	Gear	Latitude, Longitude	Depth [m]	Temperature [°C]	Salinity [‰]	No. of specimens
***Hase lagomorphicus* gen. et sp. n. (paratype CV and holotype)**	M48/1	330	17/07/2000	BC	19°06.986'S, 003°52.017'E	5468	2.4941	34.7779	1 adult ♀, 1 subadult copepodite V
***Hase lagomorphicus* gen. et sp. n. (paratype)**	M63/2	105	24/03/2005	BC	00°37.266'N, 006°28.119'W	5173	2.1087	34.5436	1 adult♀
***Hase talpamorphicus* gen. et sp. n. (paratype CV and adult)**	M85/3	1151	17/09/2011	MUC	69°05.60'N, 009°56.01'W	2270	-0.7518	34.91	1 adult ♀, 1 subadult copepodite V
***Hase talpamorphicus* gen. et sp. n. (holotype)**	M85/3	1164	18/09/2011	MUC	67°35.28'N, 006°57.48'W	2403	-0.82	34.91	adult ♀

For taxonomic studies, specimens were stained with Congo Red and mounted on slides for confocal laser scanning microscopy (CLSM) following Michels and Büntzow’s (2010) protocol. We used the following equipment and settings: Leica DCR 5000 SP5 (Leica, Wetzlar, Germany) equipped with a Leica DM 5000B microscope (Leica, Wetzlar, Germany) and three visible-light lasers (DPSS 10 mW 561 nm; HeNe 10 mW 633 nm; Ar 100 mW 458 nm, 476 nm, 488 nm and 514 nm), combined with the software LAS AF Lite, Leica Application Suite Advanced Fluorescence (Leica, Wetzlar, Germany). Series of stacked images were obtained, collecting overlapping optical sections throughout the whole preparation. Final images were obtained by maximum projection, and CLSM illustrations were composed and adjusted for contrast and brightness using the software Adobe Photoshop CS6 (Adobe Systems, San José, U.S.A.).

The habitus was drawn from whole specimens temporarily mounted in slides with glycerine, adhesive plastic discs were used to support the cover slip and prevent destruction of the specimen (Kihara and Falavigna da Rocha 2009). After CLSM microscopy, specimens were dissected under a Leica MZ12.5 (Leica, Wetzlar, Germany). Dissected parts were mounted on slides using glycerine as mounting medium, and preparations were sealed with transparent nail varnish. Drawings were made under a Leica DMR microscope equipped with Nomarsky interference contrast and a drawing tube at 400× and 1000× magnification (Leica, Wetzlar, Germany). Final illustrations were “digitally inked” using Adobe Illustrator CS6 (DIVA-1 and DIVA-2 species) or free hand inked (IceAGE species).

The terms ‘furca’ and ‘telson’ are used according to Schminke (1976). Terminology and homologisation of maxillary and maxillipedal structures follow Ferrari and Ivanenko (2008). Therefore, by the application of serial homology, the nomenclature of Huys and Boxshall (1991) for Mx2 (fig. 1.5.5, p. 26) is modified as follows: praecoxa of Mx2 is hereafter recognized as syncoxa (praecoxa and coxa), coxa is considered as the basis, and the basis is recognized as the first endopodal segment with claw. Other morphological terms are used according to Huys and Boxshall (1991).

The following abbreviations are used in the text:

**A1** antennule;

**Ae** aesthetasc;

**A2** antenna;

**enp** endopod;

**enp-1 (2,3)** proximal (middle, distal) segment of endopod;

**exp** exopod;

**exp-1 (2,3)** proximal (middle, distal) segment of exopod;

**Md** mandible;

**Mx1** maxillule;

**Mx2** maxilla;

**Mxp** maxilliped;

**P1–P6** first to sixth pereopods;

**pl** plesiomorphy;

**sy** synapomorphy.

Hyphen (i.e., “-”) between figure numbers, structures, number of spines and setae, etc. indicates all between and is inclusive (ex: P1-P5 = P1, P2, P3, P4, and P5; A-C = A, B and C; etc.)

The type material is deposited at the Forschungsinstitut und Naturmuseum Senckenberg (**SMF**) in Frankfurt, Germany.

## Taxonomy

### Order Harpacticoida Sars, 1903

#### Family Aegisthidae Giesbrecht, 1893

##### Subfamily Cerviniinae Sars M., 1903

###### Hase gen. n.

Taxon classificationAnimaliaHarpacticoidaAegisthidae

http://zoobank.org/158094D3-E3A5-427B-BFD7-1223015ABF72

####### Diagnosis.

Female body sturdy with clear distinction between prosome and narrower urosome. Prosome 5-segmented, with cephalosome and four free pedigerous somites. Cephalosome with minute spinules covering surface and anastomosing reticulation towards rostrum and along margins; posterior margin slightly serrate. Pedigerous somites with reticulation along ventroposterior margins; lateral margins of third and fourth pedigerous somites smooth or expanded posteriorly forming hook-like projections laterally; posterior margins serrate. Urosome 5-segmented, comprising P5 bearing somite, genital double-somite, two free abdominal somites, and telson. Genital double-somite and two free abdominal somites with hook-like projections ventrolaterally. Genital double-somite original segmentation indicated by transverse, serrate surface ridge with reticulation and sensilla dorsal and laterally, completely fused ventrally; genital field with copulatory pore located in median depression; gonopores covered by operculum derived from sixth legs and by anteriorly directed flap arising from somite wall; P6 fused genital opercular plate armed with two setae. Telson with well-developed anal operculum; large anal opening with folded and reticulated cuticle; surface ornamentation consisting of pair of sensilla dorsally, minute spinules and pair of pores ventrally; ventral posterior margin with minute setules. Furca symmetrical; approximately 3.4× as long as maximum width; distinctly convergent. Each ramus with seven setae: setae I-III not inserted close to each other; seta I proximal, laterally inserted, spiniform and bipinnate; seta II median, dorsally inserted, spiniform, and bipinnate; seta III subdistal, laterally inserted, spiniform and bipinnate; setae IV and V distally inserted, bipinnate and fused basally; seta VI distally inserted, minute and naked; seta VII dorsally inserted, close to seta III, tri-articulate at base and pinnate.

Rostrum fused to cephalic shield; tip rounded, with tuft of spinules along distal margin or slightly bifid and smooth; with pair of sensilla near apex. A1 7-segmented, proximal segments 1–3 cylindrical or subcylindrical; distal segments flattened. Segment I the longest; segment III with aesthetasc fused basally to single seta and set on distinct pedestal; segment VII with aesthetasc fused basally to one seta. Armature formula: I-[1], II-[8-9 elements], III-[10-12 + (1 + Ae)], IV-[3], V-[2], VI-[2], VII-[6-7 + (one naked + Ae)]. A2 3-segmented, comprising cylindrical coxa and allobasis, and 1-segmented flattened enp. Coxa small. Basis and enp-1 fused, forming elongate allobasis and with abexopodal seta. Enp medial armature four elements, apical armature 3–4 spines, one seta, and three fused elements. Exp 4-segmented; armature formula: I-[2], II-[1], III-[1], IV-[2-3].

Md. Coxa with well-developed musculature, gnathobase curved inwards, bearing several multicuspid teeth and single seta on inner distal margin. Palp well developed, comprising basis, enp and exp. Basis with four setae. Enp 1-segmented with three lateral setae and 6–7 apical setae. Exp 4-segmented; armature formula: I-[2], II-[1], III-[1], IV-[2]. Mx1. Praecoxa with row of spinules; arthrite well developed and with 13–14 elements. Coxa endite cylindrical, bearing 5–6 setae distally; epipodite absent. Basis and enp fused; basis with eleven setae; enp incorporated into basis, represented by 2–3 naked setae. Exp 1-segmented, with 2–3 setae. Mx2 comprising syncoxa fused to allobasis, and 4-segmented enp. Syncoxa/allobasis with four endites; proximal coxal endite with five pinnate setae; distal coxal endite almost completely incorporated into syncoxa, with three setae; proximal basal endite with three setae; distal basal endite with two setae and one spine. Enp-1 endite forming strong claw; accessory armature consisting of two setae, one or two spines and zero or one tube pore; armature of fused enp-2 represented by three or four elements. Free enp 3-segmented; armature formula: I-[claw; 3–4 spines/setae; 0–1 tube pore], II-[3-4], III-[2], IV-[2-3], V-[3-4]. Mxp with elongated syncoxa, strong basis and 2-segmented enp; syncoxal endites represented proximal to distal by two elements, 3–4 elements, and 2–3 elements; basal endite represented by two elements. Enp with armature formula: I-[2], II-[four elements].

Pereopods biramous; exp and enp flattened, bent inwards, especially on P1 and P2. Praecoxa without ornamentation. Coxa without ornamentation (P1) or ornamented (P2-P4). Basis with (P1 and P2) or without (P3 and P4) one seta on outer proximal corner, with one seta on inner distal corner of P1. Exp 3-segmented. Enp 3-segmented (P1), 2-segmented (P2 and P3) and 1-segmented (P4). P5 1-segmented, pointing outwards, fused to supporting somite. Exp with three elements. P1-P4 spine and setal formulae as follows:

**Table:** 

	Exp	Enp
P1	I, 1; I, 1; II, II+1, 1	0, 1; 0, 1; I, 2, 2
P2	I, 1; I, 1; II, II+1, 2	0, 1; I, 2, 1
P3	I, 1; I, 1; II, II+1, 2	0, 1; I, 2, 0
P4	I, 1; I, 1; II, II+1, I-II	0, 2, 0-I

####### Etymology.

The generic name, *Hase*, from German, means “hare”, and refers to the very superficial resemblance of the new species to a hare or rabbit. Gender masculine.

####### Type species.

*Hase lagomorphicus* sp. n., by present designation.

###### Hase lagomorphicus

Taxon classificationAnimaliaHarpacticoidaAegisthidae

gen. et sp. n.

http://zoobank.org/582DC8A7-6041-44AE-85EC-AEB38ED60BBC

Figs 2 , 3 , 4 , 5 , 6 , 7 , 8 , 9 , 10 , 11

####### Type material.

Holotype, adult female dissected on six slides (reg. no. SMF 37130/1-6), from DIVA-1 (M48/1, 330). Paratype, adult female (incomplete) dissected into three slides (reg. no. SMF 37131/1-3), from DIVA-2 (M63/2, 105). Paratype 2, subadult copepopid stage V (CV) dissected into five slides (reg. no. SMF 37132/1-5), from DIVA-1 (M48/1, 330).

####### Type locality.

Angola Basin (DIVA-1 cruise M48/1, 330) (Fig. 1; Table 1), Atlantic Ocean.

####### Etymology.

The specific epithet is built by combining the ancient Greek lexemes λαγός (lagós), meaning hare, and μορφώ (morphó), “the Shapely One”.

####### Description.

Female. Total body length 730 μm (paratype 1) and 735 μm (holotype) (*N* = 2; mean = 732.5 μm). Largest width measured at posterior margin of P2-bearing somite: 292 μm (paratype 1) and 295 μm (holotype) (*N* = 2; mean = 293.5 μm).

**Figure 2.**
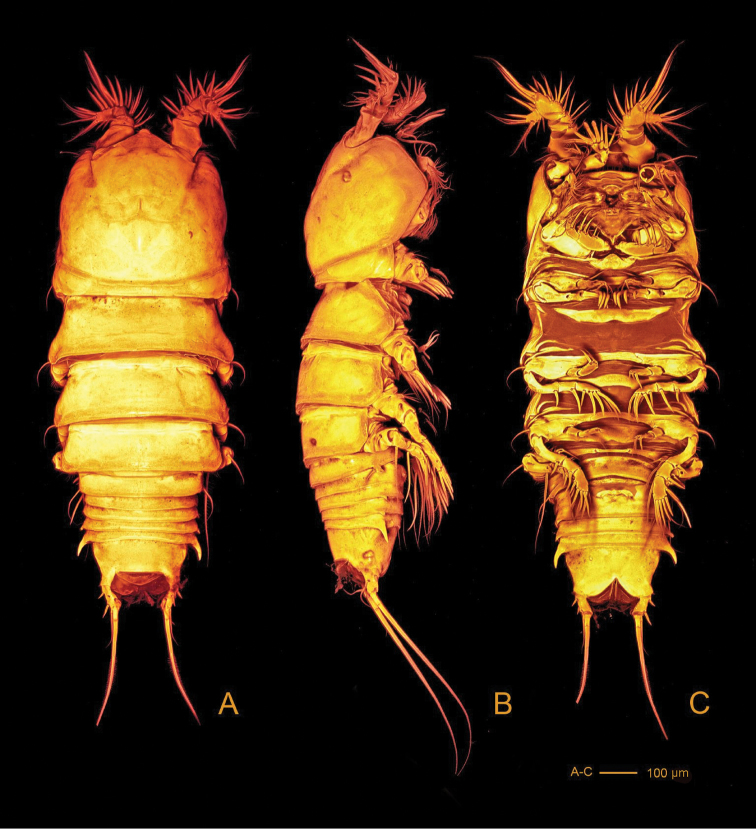
*Hase lagomorphicus* gen. et sp. n. Confocal laser scanning images. Holotype (female) (M48/1, 330, DIVA-I): **A** habitus, dorsal **B** habitus, lateral **C** habitus, ventral.

*Body* (Fig. 2A–C) with clear distinction between prosome and narrower urosome. Prosome (Fig. 2A–C) 5-segmented, with cephalosome and P1-P4 free pedigerous somites. Cephalosome with spinules covering surface and anastomosing reticulation towards rostrum and along margins; posterior margin slightly serrate. Pedigerous somites with reticulation along ventroposterior margins (Fig. 2B); lateral margins of third and fourth pedigerous somites smooth (Fig. 2A, B); posterior margins serrate.

**Figure 3.**
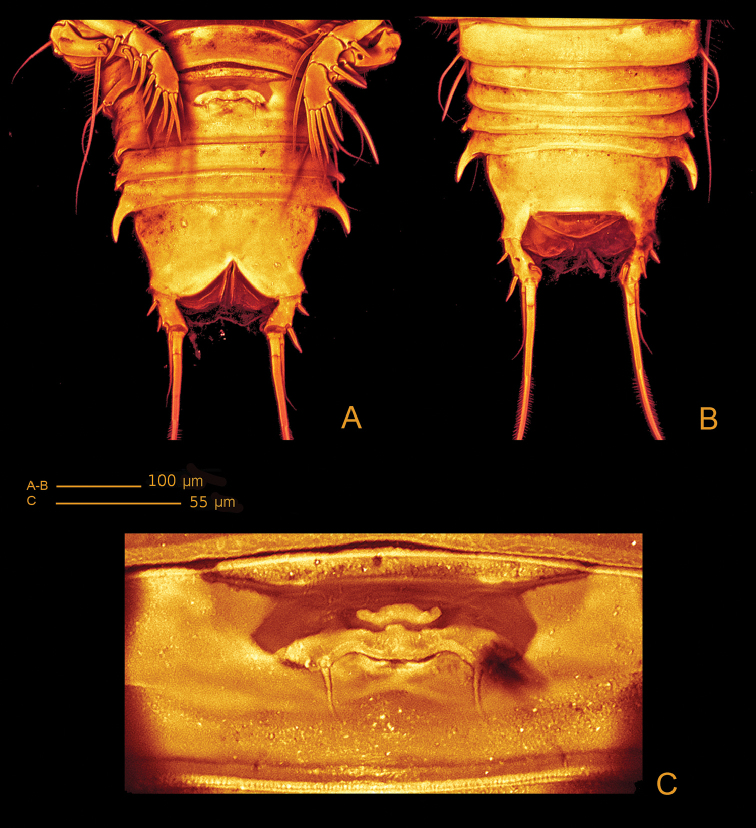
*Hase lagomorphicus* gen. et sp. n. Holotype (female) (M48/1, 330, DIVA-I): **A** urosome, ventral **B** urosome, dorsal **C** genital double somite, ventral.

*Urosome* (Figs 2A–C, 3A, B) 5-segmented, comprising P5-bearing somite, genital double-somite, two free abdominal somites and telson. Genital double-somite and two free abdominal somites with hook-like projections ventrolaterally, distalmost the largest.

**Figure 4.**
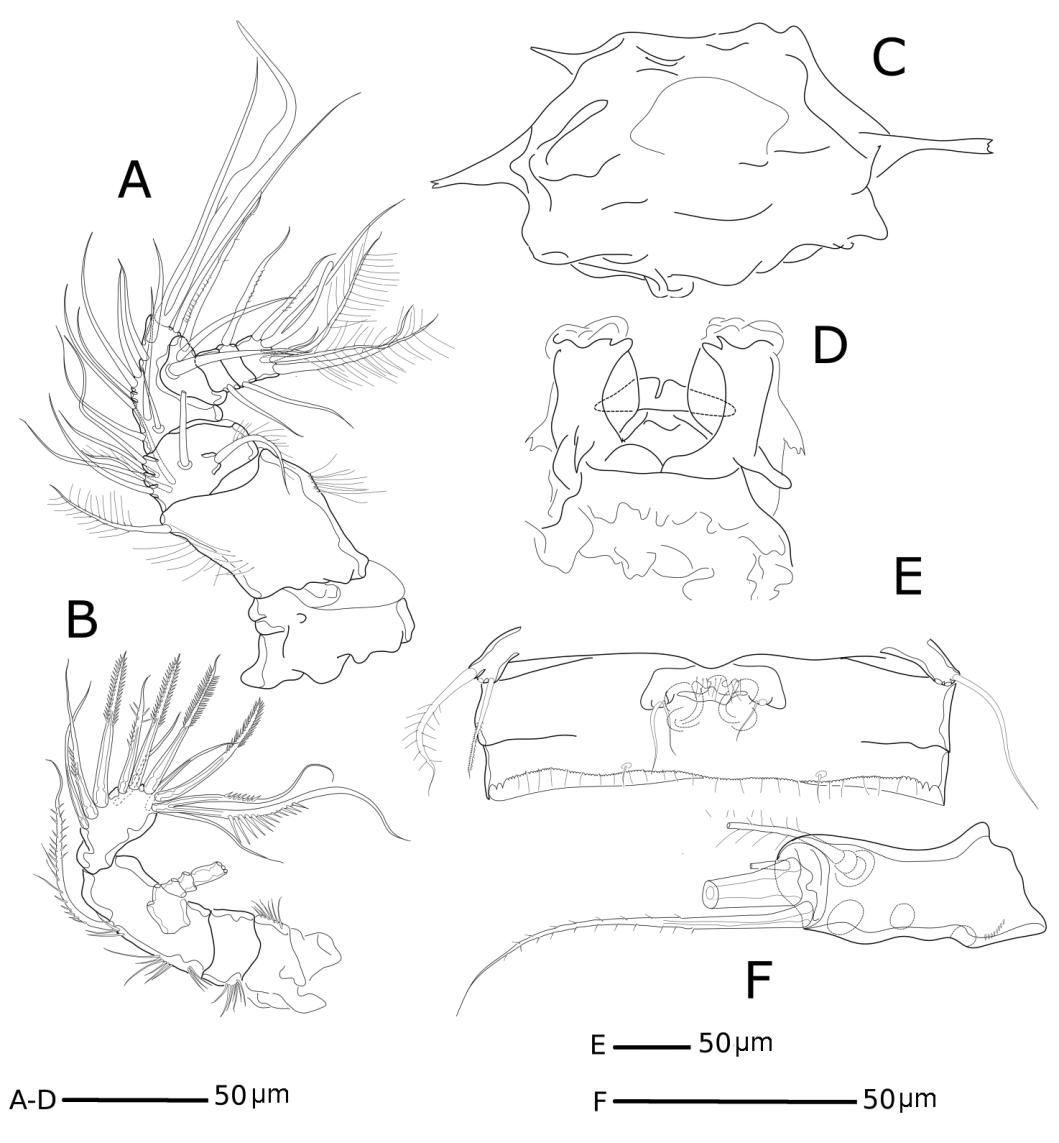
*Hase lagomorphicus* gen. et sp. n. Holotype (female) (M48/1, 330, DIVA-I): **A** A1 **B** A2 **C** labrum **D** labium **E** P5 and genital double somite **F** furca.

*Genital double-somite* (Figs 2B, C, 3A–C, 4E) original segmentation indicated by transverse surface ridge with reticulation and sensilla dorsal and laterally, completely fused ventrally; genital field (Figs 3C, 4E) with copulatory pore slightly covered by a proximal flap, pointing posteriorly, located in a soft median depression; gonopores covered by operculum derived from sixth legs and anteriorly directed flap, medially depressed, arising from somite wall; P6 bearing two naked seta.

*Telson* (Figs 2A–C, 3A, B) with well-developed anal operculum; large anal opening with folded and reticulated cuticle; surface ornamentation consisting of pair of sensilla dorsally, minute spinules and pair of pores ventrally; ventral posterior margin with minute setules.

*Furca* (Figs 2A–C, 3A, B, 4F) symmetrical; approximately 3.4× as long as maximum width; distinctly convergent. Each ramus with seven setae: seta I, spiniform and bipinnate, inserted laterally, close to proximal margin; seta II, spiniform and bipinnate, dorsal and medially inserted; seta III laterally inserted, spiniform and bipinnate, located at outer subdistal corner; setae IV and V distally inserted, fused basally, seta IV bipinnate, seta V bipinnate and 4× longer than seta IV; seta VI distally inserted, minute and naked; seta VII dorsal, close to seta III, tri-articulate at base and pinnate.

*Rostrum* (Fig. 2A) fused to cephalic shield; tip rounded, with tuft of spinules along distal margin; with pair of sensilla near apex.

**Figure 5.**
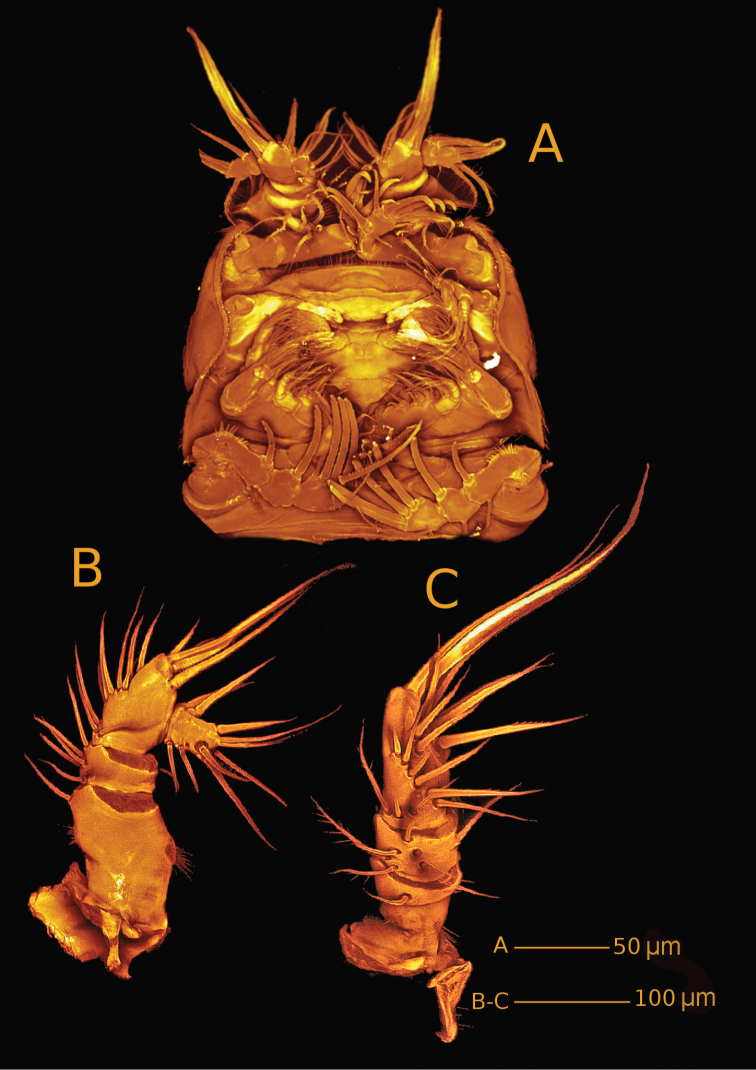
*Hase lagomorphicus* gen. et sp. n. Confocal laser scanning images. Holotype (female) (M48/1, 330, DIVA-I): **A** cephalothorax and first pedigerous somite, ventral **B** A1, dorsal **C** A1, inner.

*A1* (Figs 4A, 5B, C) 7-segmented, proximal segments 1–3 cylindrical or subcylindrical; distal segments flattened. Segment I the longest, with rows of setules along outer and inner margins; segment III with aesthetasc fused basally to seta and set on distinct pedestal; segment VII with aesthetasc fused basally to one naked seta.

Armature formula: I-[one pinnate], II-[six naked + two unipinnate], III-[ten naked + (one naked + Ae)], IV-[one bipinnate + two naked], V-[two naked], VI-[one unipinnate + one naked], VII-[two naked, three bipinnate + one unipinnate + (one naked + Ae)].

*A2* (Figs 4B, 7A) 3-segmented, comprising cylindrical coxa and allobasis, and 1-segmented and flattened enp. Coxa small, with spinules along inner margin. Basis and enp-1 fused, forming elongate allobasis, with patches of spinules as shown; abexopodal seta long and bipinnate. Medial armature of free enp consisting of two smooth setae, one seta medially unipinnate, one distally bipinnate spine and one seta medially unipinnate and distally bipinnate; apical armature consisting of three bipinnate spines, one naked seta and three elements fused basally (two long setae medially unipinnate, and one smooth). Exp 4-segmented; distal segment with row of spinules; armature formula: I-[two pinnate], II-[one pinnate], III-[one pinnate], IV-[two pinnate].

**Figure 6.**
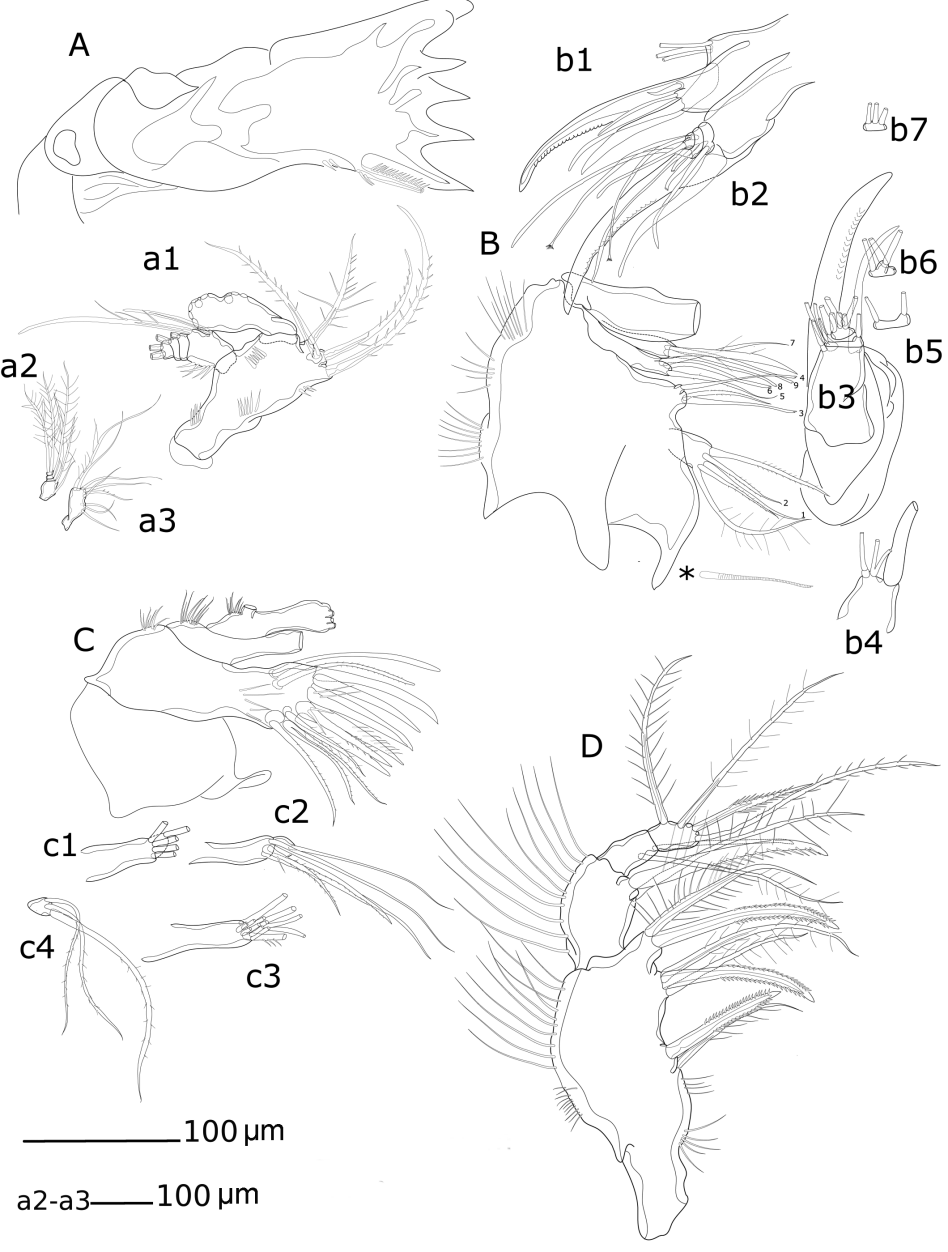
*Hase lagomorphicus* gen. et sp. n. Holotype (female) (M48/1, 330, DIVA-I): **A** Md gnathobasis; a1- mandibular palp with basis, exp and enp; a2- exp; a3- enp **B** Mx2 syncoxa, allobasis, and first enp without claw; b1 enp1 with claw and accessory spines, and 2^nd^ enp (fused) in lateral view; b2 enp-1 with claw, and enp-2 to enp-5; b3 upper view of enp-1 to enp-5; b4 upper view of enp-2; b5-b7 upper view of enp-3 to enp-5 **C** Mx1 with unarmed coxa, basis, enp and exp; c1 and c2- coxa; c3 basis with incorporated enp; c4- exopod **D** Mxp.

*Md* (Fig. 6A(a1, a2, a3), 7D, E). Coxa with well-developed musculature, gnathobase curved inwards, bearing several multicuspid teeth and one bipinnate seta on inner distal margin; two rows of spinules near insertion area of bipinnate seta. Palp well developed, comprising basis, enp and exp. Basis with four bipinnate setae and surface ornamentation as indicated in Fig. 6A (a1). Enp 1-segmented with three smooth lateral setae and six apical setae (four naked and two unipinnate). Exp 4-segmented, exp-1 as long as next three segments combined; armature formula: I-[one smooth and one bipinnate], II-[one bipinnate], III-[one bipinnate], IV-[two bipinnate].

**Figure 7.**
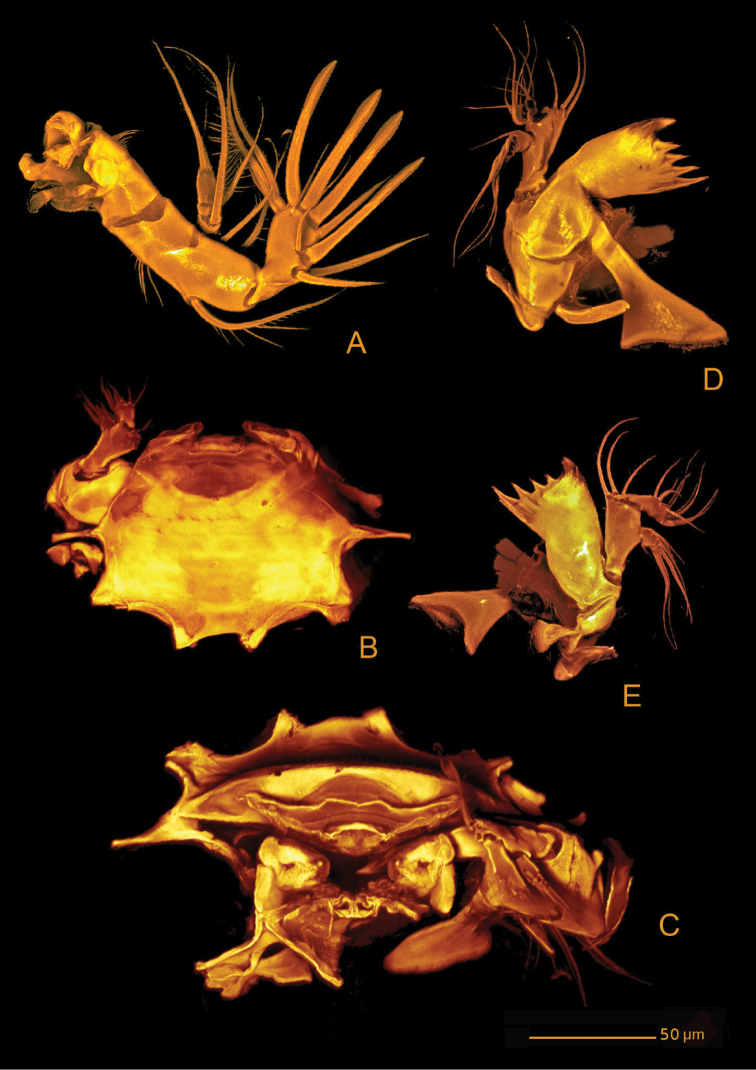
*Hase lagomorphicus* gen. et sp. n. Confocal laser scanning images. Holotype (female) (M48/1, 330, DIVA-I): **A** A2 **B** labrum, anterior **C** labrum, labium and Md, ventral **D** Md, anterior **E** Md, posterior.

*Mx1* (Figs 6C(c1–c4), 8A, B). Praecoxa with row of spinules; arthrite well developed, with one pinnate and one smooth seta on anterior surface, four smooth spines, and three pinnate spines along distal margin (two ornate with two large spinules at basis), four pinnate setae on aboral margin, two fused at basis. Coxa endite cylindrical, bearing five setae (four naked and one pinnate) distally; epipodite absent. Basis and enp fused; basis with eleven setae; enp incorporated into basis, represented by two naked setae. Exp 1-segmented, with three bipinnate setae.

**Figure 8.**
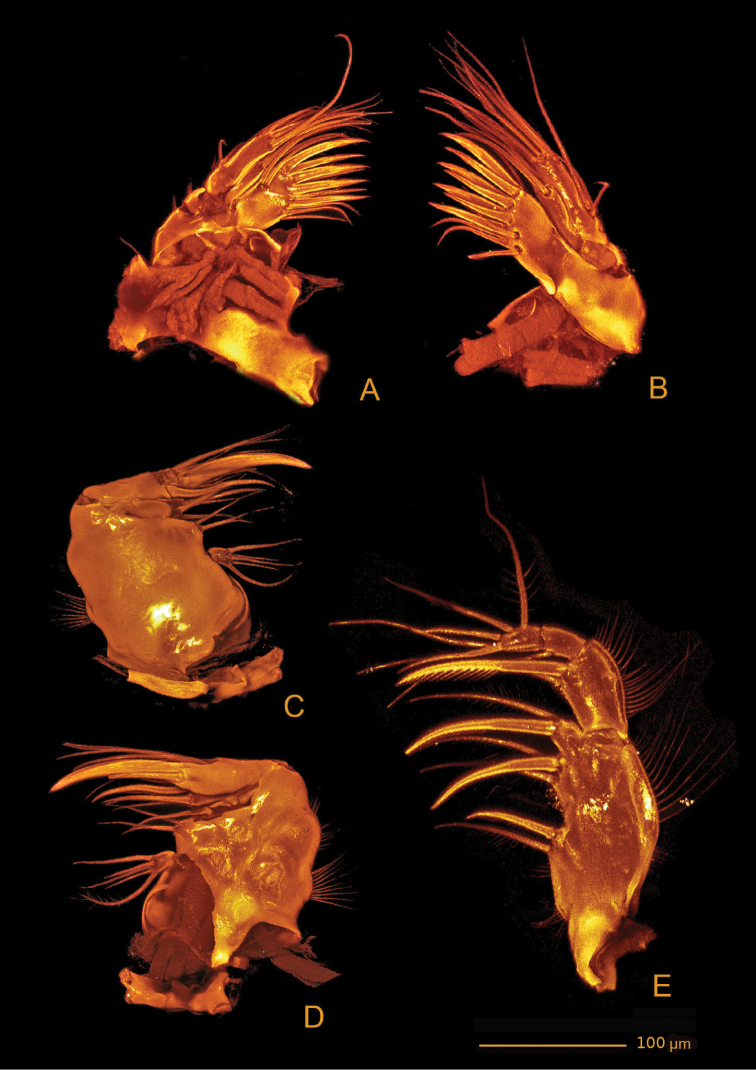
*Hase lagomorphicus* gen. et sp. n. Confocal laser scanning images. Holotype (female) (M48/1, 330, DIVA-I): **A** Mx1, posterior **B** Mx1, anterior **C** Mx2, anterior **D** Mx2, posterior **E** Mp, anterior.

*Mx2* (Figs 6B(b1–b7), 8C, D) comprising syncoxa fused to allobasis, and 5-segmented enp. Syncoxa/allobasis with four endites; proximal coxal endite with five pinnate setae; distal coxal endite almost completely incorporated into syncoxa, with one pinnate setae, and two naked setae with bifid tip; proximal basal endite with three setae (two naked, one with bifid tip, and one weakly pinnate); distal basal endite with two naked setae with bifid tip, and one weakly pinnate spine. Enp-1 endite forming strong claw; accessory armature consisting of two naked setae (one long and flexible and one foliaceous), one spine and one claw-like spine; armature of fused enp-2 represented by three naked seta. Free enp 3-segmented; armature formula: I-[claw; 4], II-[3], III-[2], IV-[3], V-[3].

*Mxp* (Figs 6D, 8E) with elongated syncoxa, strong basis, and 2-segmented enp. Syncoxa with rows of spinules along inner and outer margins; syncoxa with three endites; first endite with one bipinnate seta and one bipinnate spine; second endite with two bipinnate setae and one bipinnate spine; third endite with one bipinnate seta and one bipinnate spine; basal endite with one bipinnate seta and one unipinnate spine. Enp with armature formula: I-[two setae; one bipinnate and one naked], II-[one unipinnate spine + three bipinnate setae].

**Figure 9.**
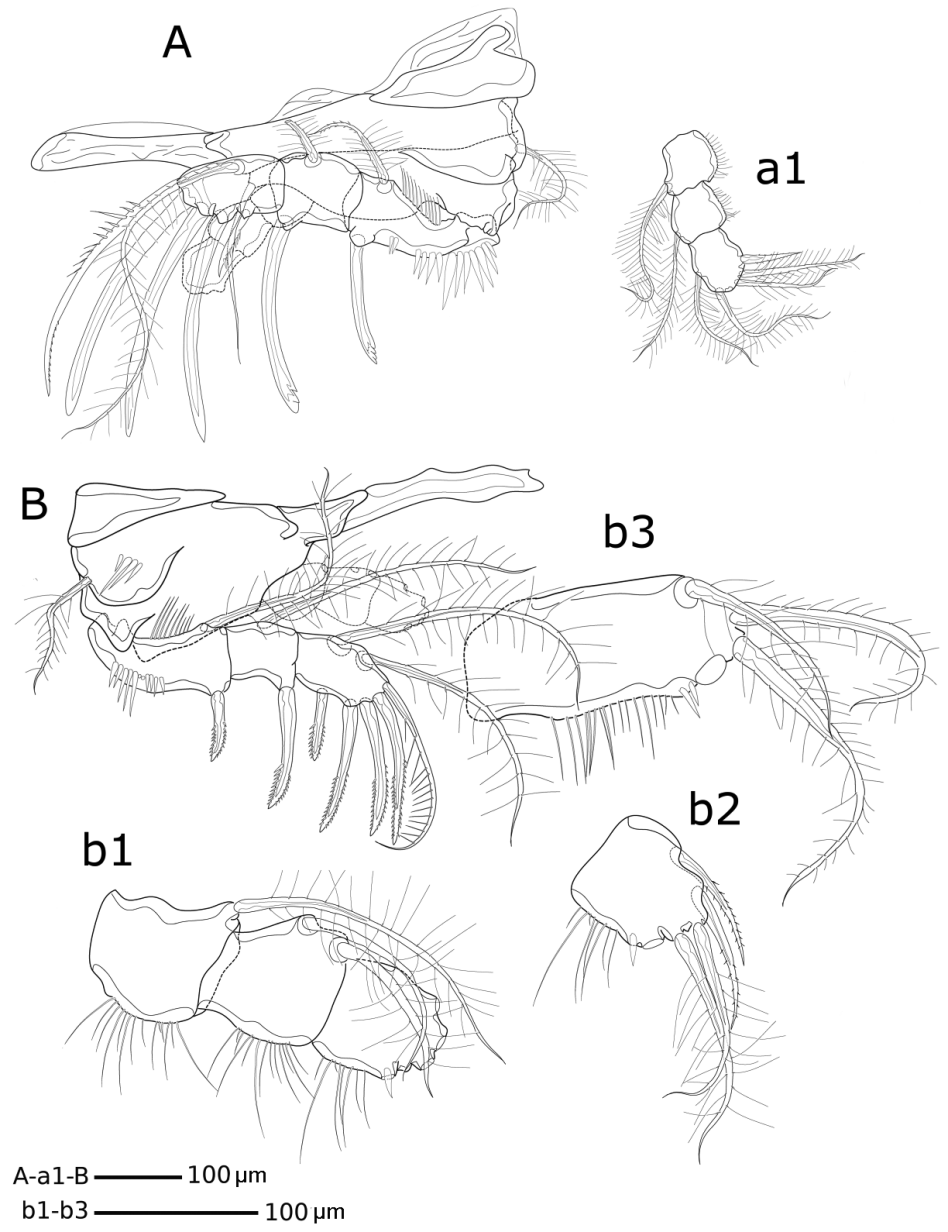
*Hase lagomorphicus* gen. et sp. n. Holotype (female) (M48/1, 330, DIVA-I): **A** P1; a1- P1 enp.; b3- P2 enp-2. Paratype (Copepodite V) (M48/1, 330, DIVA-I) **B** P2; b1- P2 enp; b2- P2 enp-3.

**Figure 10.**
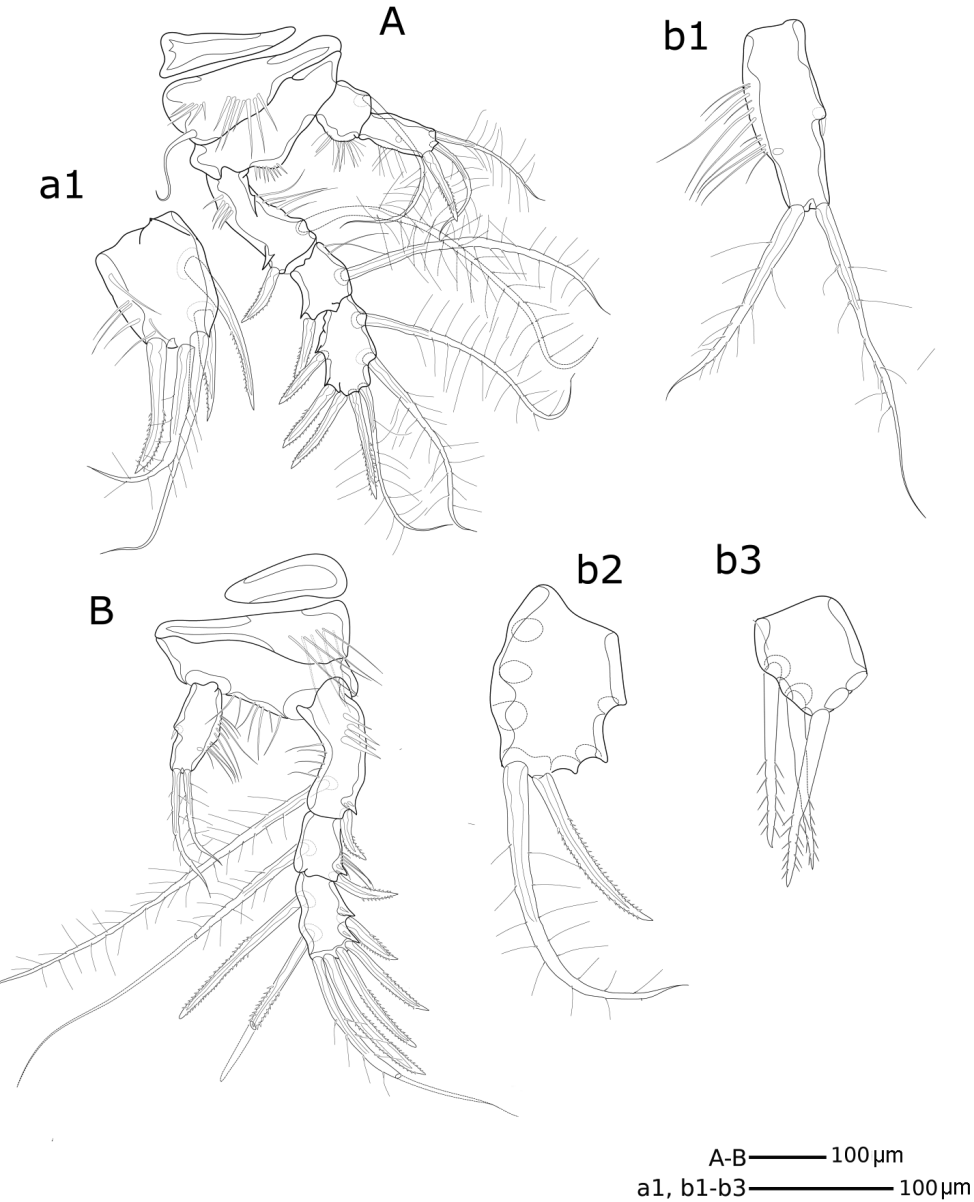
*Hase lagomorphicus* gen. et sp. n. Holotype (female) (M48/1, 330, DIVA-I): **A** P3 **B** P4; b1- P4 enp. Paratype (Copepodite V) (M48/1, 330, DIVA-I) : a1- P3 enp-3; b2- P4 exp-3; b3- P4 enp-3.

**Figure 11.**
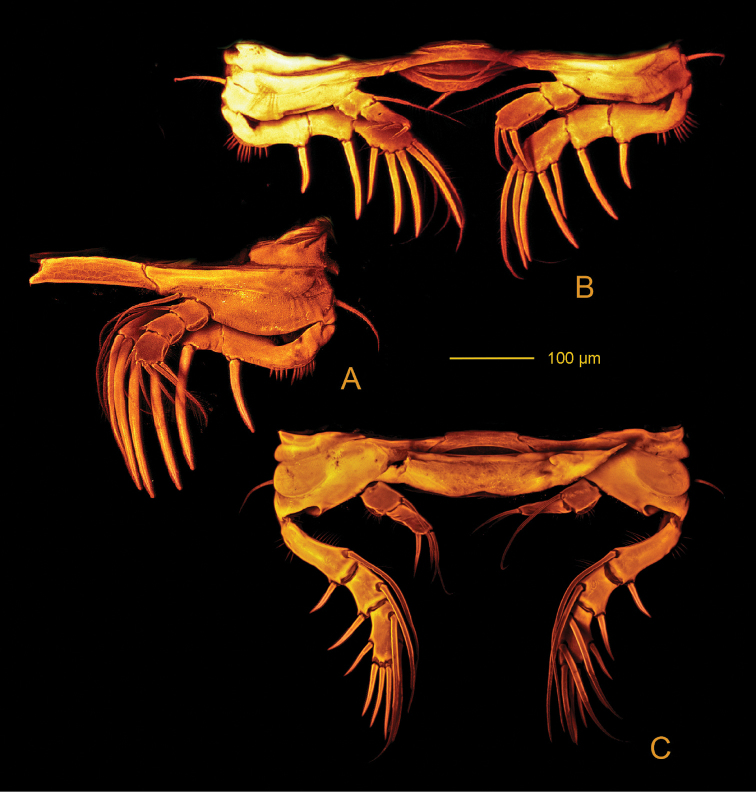
*Hase lagomorphicus* gen. et sp. n. Confocal laser scanning images. Holotype (female) (M48/1, 330, DIVA-I): **A** P1 **C** P3. Paratype (female) (M63/2, 105, Diva II) **B** P2.

Pereopods (Figs 5A, 9–11) biramous; exp and enp bent inwards, especially on P1 and P2. Praecoxa transversally elongate, without ornamentation. Coxa without ornamentation (P1) or ornamented (P2-P4), with position and strength of ornamentation differing from P2 to P4. Basis with (P1, P2) or without (P3, P4) bipinnate seta on outer proximal corner, with bipinnate seta on inner distal corner of P1. Exp 3-segmented; bent inwards against basis in P1 and P2, exp-1 with rows of setules along inner margin and spinules along outer margin, exp-2 without ornamentation on P1 and P2, with setules on inner margin of P3 and outer margin of P4. Enp 3-segmented on P1, 2-segmented on P2 and P3 but 1-segmented on P4; enp-1 with setules along outer margin of P1-P3; enp P4 with setules on outer margin. Setal formulae as follows:

**Table:** 

	Exp	Enp
P1	I, 1; I, 1; II, II+1, 1	0, 1; 0, 1; I, 2, 2
P2	I, 1; I, 1; II, II+1, 2	0, 1; I (broken), 2, 1
P3	I, 1; I, 1; II, II+1, 2	0, 1; I, 2, 0
P4	I, 1; I, 1; II, II+1, II	0, 2, I

P5 (Fig. 4E) One-segmented, fused to supporting somite, pointing outwards. Exp with three elements (one lost during dissection), outer most a bipinnate seta, innermost a bipinnate spine.

Male unknown.

####### Occurrence.

Angola and Guinea basins, Atlantic Ocean.

####### Remarks.

In the subadult CV, enp is 3-segmented on P2-P4; exp-3 of P4 with 8 elements (Fig. 10 b2); enp-2 of P2-P4 with two inner setae (Fig. 9 B, b1), exp-3 of P2-P4 with 5 elements (Fig. 9 b3 and Fig. 10 (a1, b3)). Setal formulae as follows:

**Table:** 

	Exp	Enp
P1	I, 1; I, 1; II, II+1, 1	0, 1; 0, 1; I, 2, 2
P2	I, 1; I, 1; II, II+1, 2	0, 1; 0, 2; 0, I+2 (?), II
P3	I, 1; I, 1; II, II+1, 2	0, 1; 0,2; 0, I+2, II
P4	I, 1; I, 1; II (?), II (?) +1, 3 elements (broken)	0, 1; 0, 2; 5 elements (two broken)

###### Hase talpamorphicus

Taxon classificationAnimaliaHarpacticoidaAegisthidae

gen. et sp. n.

http://zoobank.org/E1475D7D-08B2-4E01-A1DE-3E849E71C2DB

Figs 12 , 13 , 14 , 15 , 16 , 17 , 18 , 19 , 20

####### Type material.

Holotype female dissected on 21 slides (reg. no. SMF 37133/1-21) from station 1164, multi corer 9. Undissected paratypes: one female (reg. no. SMF 37134/1) from station 1151, MUC 12 and one subadult copepopid stage V (CV) (reg. no. SMF 37135/1) from station 1151, MUC 10. All specimens were collected during the Overflow, Circulation and Biodiversity Expedition of the RV “Meteor” (Cruise No. M85/3).

####### Type locality.

Norwegian Sea (IceAGE cruise M85/3, 1164) (Fig. 1; Table 1).

####### Etymology.

The specific epithet is built by combining the Latin *talpa*, meaning a mole, and the ancient Greek lexeme *μορφώ* (*morphó*), “the Shapely One”.

####### Description.

Female. Total body length 986.7 μm (holotype) and 1000.0 μm (paratype) (*N* = 2; mean = 993.4 μm). Largest width measured at posterior margin of P2-bearing somite: 400.0 μm (holotype) and 437.5 μm (paratype) (*N* = 2; mean = 418.7 μm).

**Figure 12.**
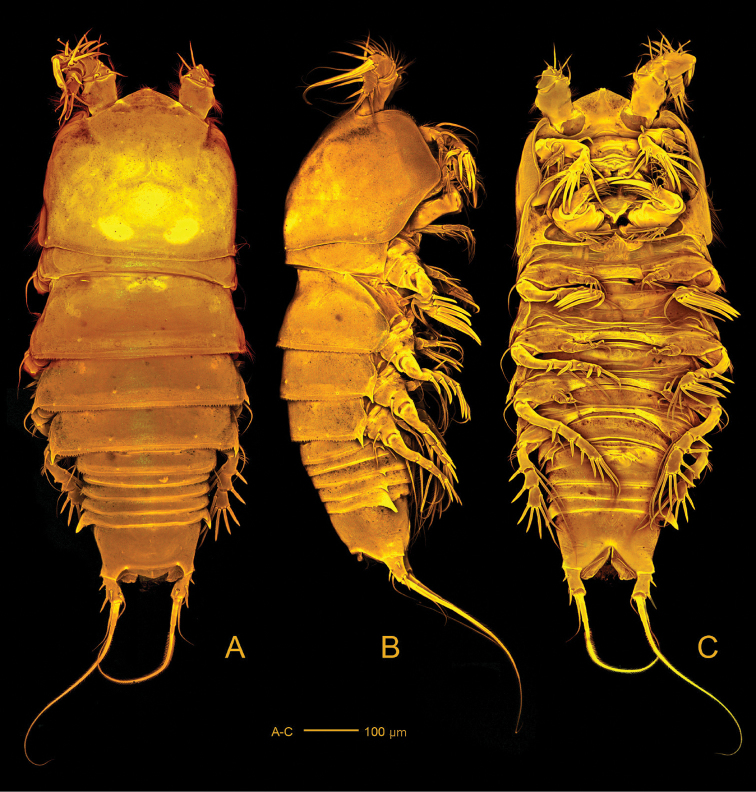
*Hase talpamorphicus* gen. et sp. n. Confocal laser scanning images. Holotype (female) (M85/3, 1164): **A** habitus, dorsal **B** habitus, lateral **C** habitus, ventral.

*Body* (Fig. 12A–C) with clear distinction between prosome and narrower urosome. Prosome 5-segmented, with cephalosome and four free pedigerous somites. Cephalosome with minute spinules covering surface and anastomosing reticulation towards rostrum and along margins; additional ornamentation consisting of sensilla and pores; posterior margin slightly serrate. Pedigerous somites with reticulation along posterior margins and ornamentation consisting of sensilla; lateral margins of third and fourth pedigerous somites expanded posteriorly forming hook-like projections laterally; posterior margins serrate.

**Figure 13.**
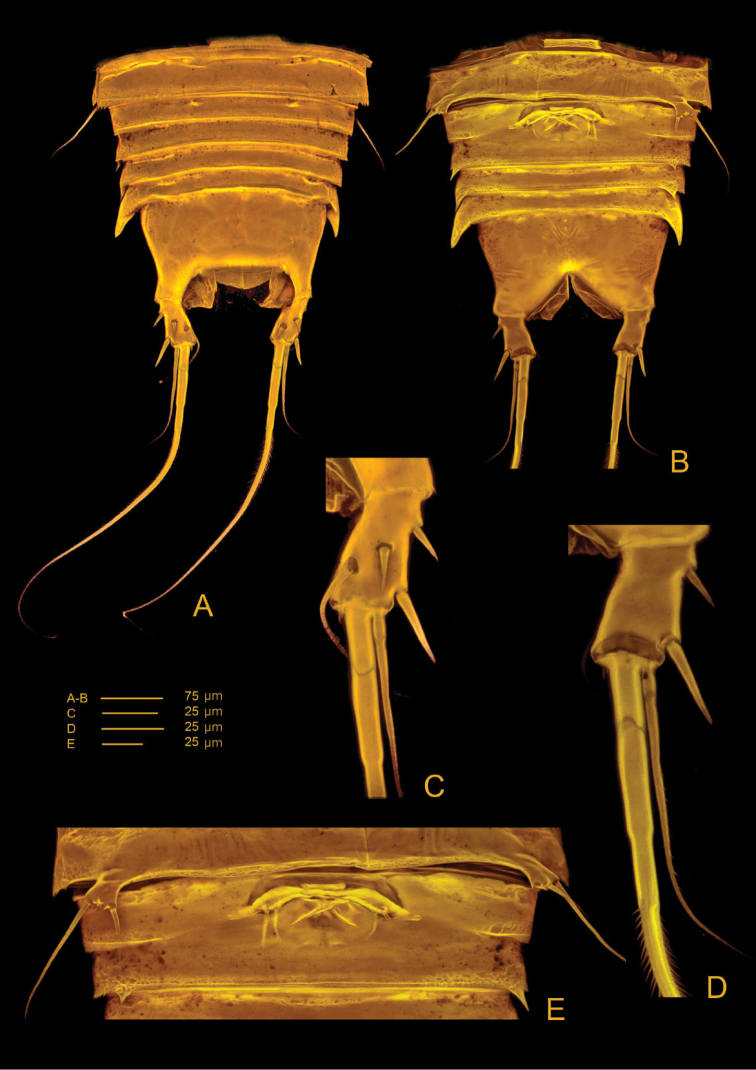
*Hase talpamorphicus* gen. et sp. n. Confocal laser scanning images. Holotype (female) (M85/3, 1164): **A** urosome, dorsal **B** urosome, ventral **C** furca, dorsal **D** furca, ventral **E** P5 and double genital somite.

*Urosome* (Figs 12A–C, 13A, B) 5-segmented, comprising P5-bearing somite, genital double-somite, two free abdominal somites, and telson. Urosomites with surface ornamentation consisting of sensilla and minute spinules, spinules more conspicuous ventrally; posterior margin serrate and with reticulated surface, genital double-somite and two free abdominal somites with hook-like projections ventrolaterally, larger in somite anterior to telson.

*Genital double-somite* (Figs 12C, 13B, E, 19D) original segmentation indicated by transverse, serrate surface ridge with reticulation and sensilla dorsal and laterally, completely fused ventrally; genital field (Figs 12C, 13B, E, 19D) with copulatory pore completely visible, not covered by a proximal flap as observed for the previous species, located in a well-developed median depression; gonopores covered by operculum derived from sixth legs and by anteriorly directed and straight flap arising from somite wall; P6 bearing two naked setae.

*Telson* (Figs 12A–C, 13A, B) with well-developed anal operculum; large anal opening with folded and reticulated cuticle; surface ornamentation consisting of pair of sensilla dorsally, minute spinules and pair of pores ventrally; ventral posterior margin with minute setules.

**Figure 14.**
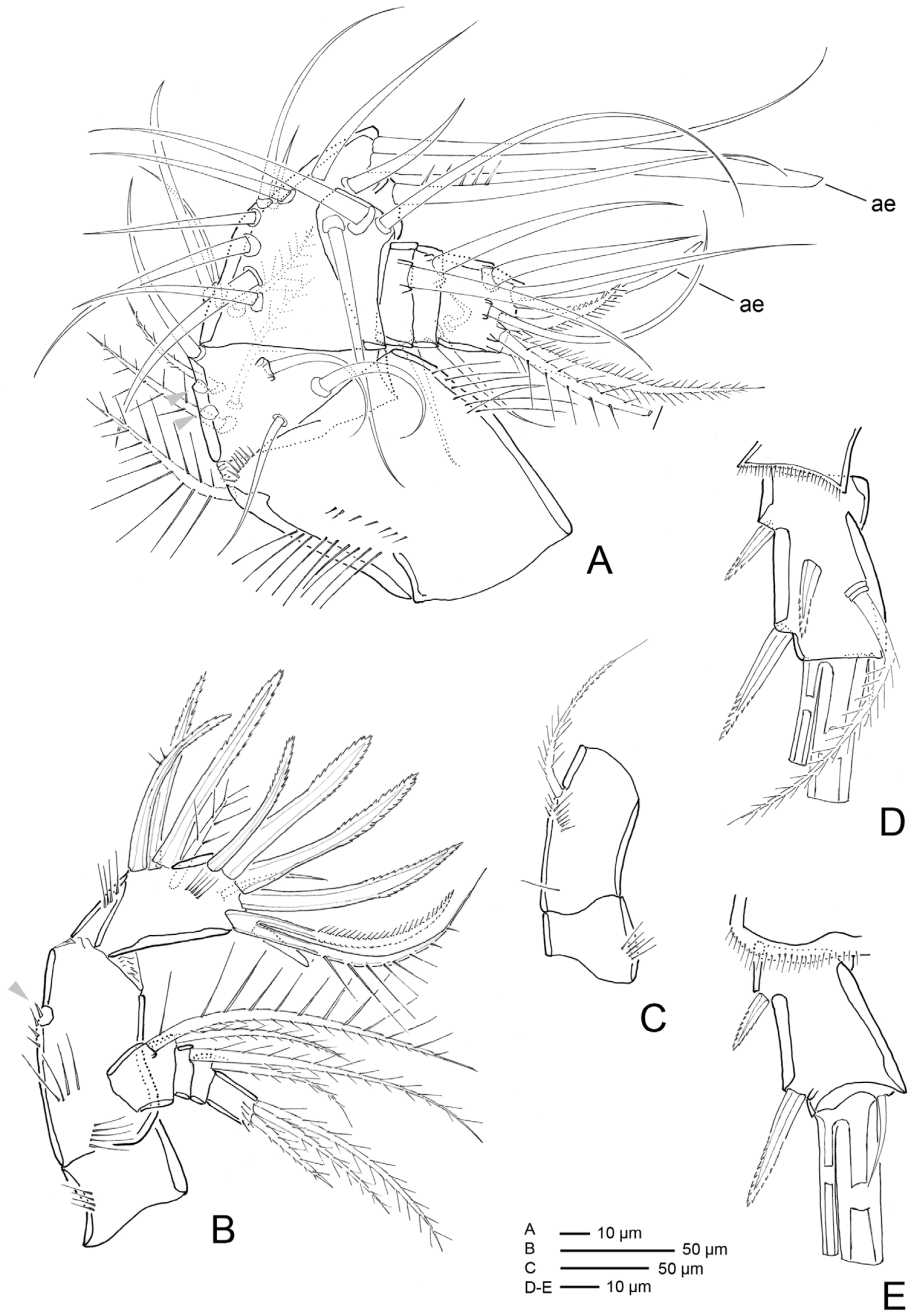
*Hase talpamorphicus* gen. et sp. n. Holotype (female) (M85/3, 1164): **A** antennule **B** A2 **C** A2 coxa and allobasis **D** furca, dorsal **E** furca, ventral.

*Furca* (Figs 12A–C, 13A–D, 14D, E) symmetrical; approximately 3.4× as long as maximum width; distinctly convergent. Each ramus with seven setae: seta I, spiniform and bipinnate, close to anterior margin; seta II, spiniform and bipinnate, located dorsally; seta III spiniform and bipinnate, located at outer distal corner; setae IV and V fused basally, seta IV bipinnate, seta V bipinnate and 4× longer than seta IV; seta VI minute and naked; seta VII tri-articulate at base and pinnate.

*Rostrum* (Fig. 12A, C) fused to cephalic shield; tip slightly bifid; with pair of sensilla and midventral tube-pore near apex.

**Figure 15.**
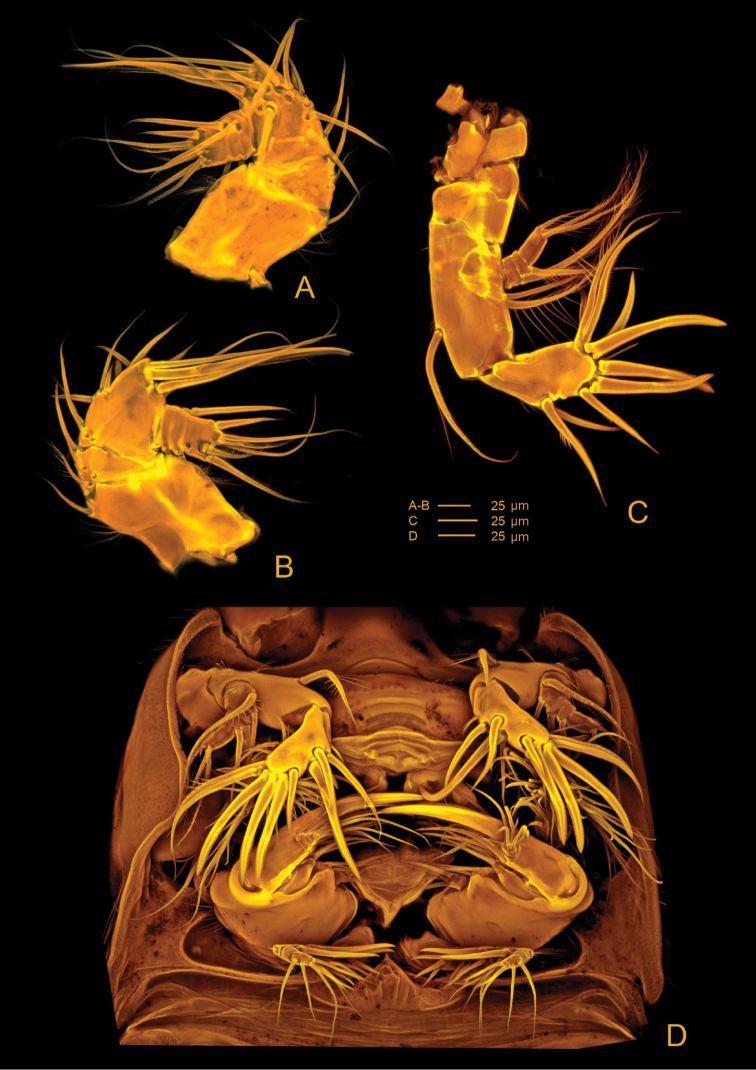
*Hase talpamorphicus* gen. et sp. n. Confocal laser scanning images. Holotype (female) (M85/3, 1164): **A** A1, ventral **B** A1, dorsal **C** A2 **D** ventral cephalothorax showing A2 and mouthparts.

*A1* (Figs 14A, 15A, B) 7-segmented. Shape as in previous species. Segment I the longest, with rows of setules along outer and inner margins, with small spinules along outer distal corner; segment III with aesthetasc fused basally to seta and set on distinct pedestal; segment VII with aesthetasc fused basally to one naked seta.

Armature formula: I-[one pinnate], II-[four naked + three bipinnate + two missing elements], III-[eleven naked + one bipinnate + (one naked + ae)], IV- [three naked], V-[two naked], VI-[two naked], VII- [three naked, three pinnate + (one naked + ae)].

*A2* (Figs 14B, C, 15C, D) 3-segmented, comprising cylindrical coxa and allobasis, and flattened 1-segmented enp. Coxa small, with spinules along inner margin. Basis and enp-1 fused, forming elongate allobasis, with denticles along abexopodal margin and patch of spinules; abexopodal seta bipinnate. Free enp ornamented with rows of spinules on anterior surface; medial armature consisting of three pectinate spines and one bipinnate seta; apical armature consisting of four pectinate spines, one naked seta and three elements fused basally (one bipinnate seta, one unipinnate seta and one small flattened seta). Exp 4-segmented; distal segment with row of spinules; armature formula: I-[two pinnate], II-[one pinnate], III-[one pinnate], IV-[three pinnate].

**Figure 16.**
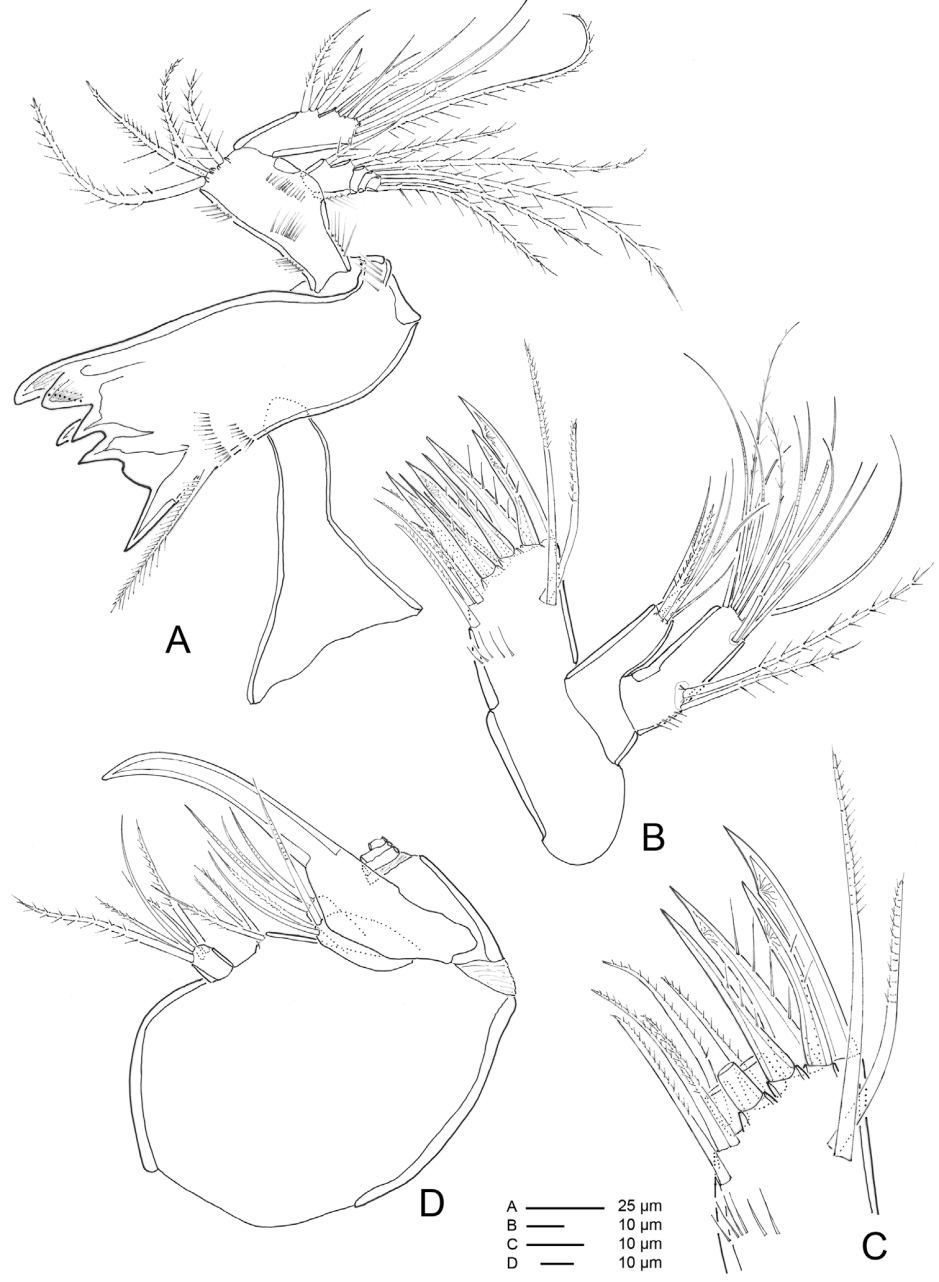
*Hase talpamorphicus* gen. et sp. n. Holotype (female) (M85/3, 1164): **A** mandible **B** Mx1 **C** Mx1 praecoxal arthrite **D** Mx2.

**Figure 17.**
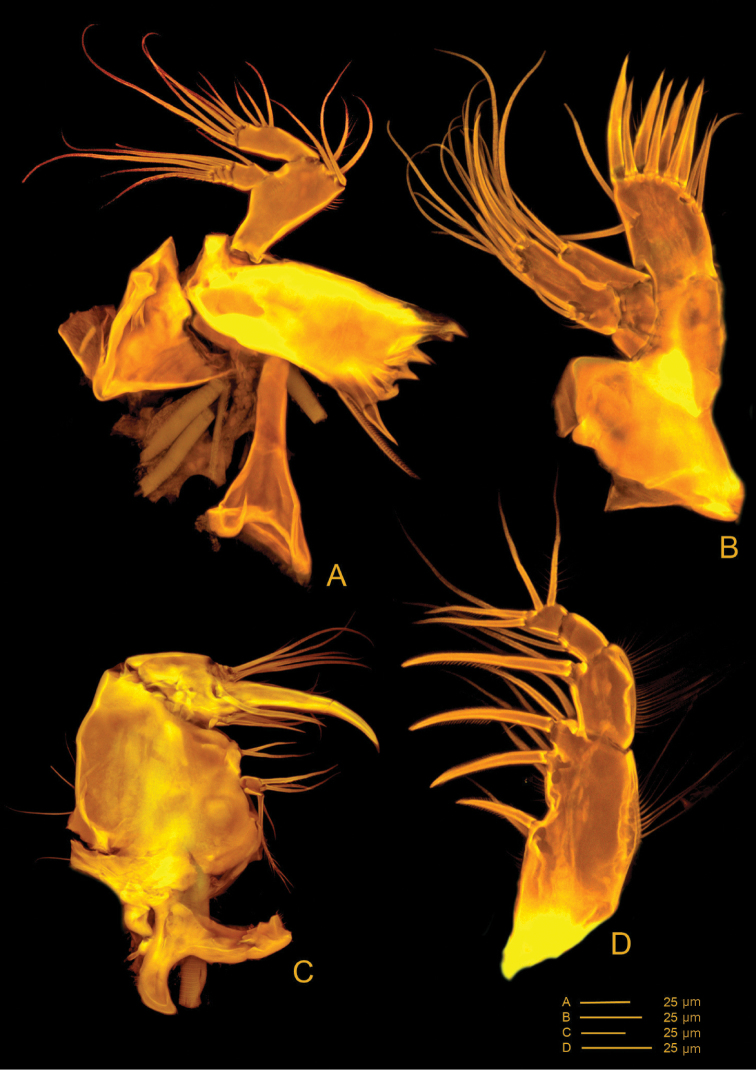
*Hase talpamorphicus* gen. et sp. n. Confocal laser scanning images. Holotype (female) (M85/3, 1164): **A** Md, anterior **B** Mx1 **C** Mx2 **D** Mxp.

*Md* (Figs 16A, 17A). Coxa with well-developed musculature, gnathobase curved inwards, with several multicuspidate teeth and one bipinnate seta on inner distal margin; rows of spinules near insertion area of bipinnate seta. Palp well developed, with basis, enp and exp. Basis with four bipinnate setae and surface ornamentation as indicated in Figure 14A. Enp 1-segmented with three lateral setae (two bipinnate and one unipinnate) and seven apical setae (four naked, two bipinnate and one unipinnate). Exp 4-segmented, exp-1 as long as next three segments combined; armature formula: I-[two bipinnate], II-[one bipinnate], III-[one bipinnate], IV-[two bipinnate].

*Mx1* (Figs 16B, C, 17B). Praecoxa with row of spinules as shown; arthrite well developed, with two pinnate setae on anterior surface, seven pinnate and striated spines and three bipinnate setae along distal margin, two bipinnate setae on posterior surface. Coxa endite cylindrical, bearing six setae (five naked and one pinnate) distally; epipodite absent. Basis and enp fused; basis with eleven setae (nine naked and two bipinnate); enp incorporated into basis, represented by three naked setae. Exp 1-segmented, with two bipinnate setae.

**Figure 18.**
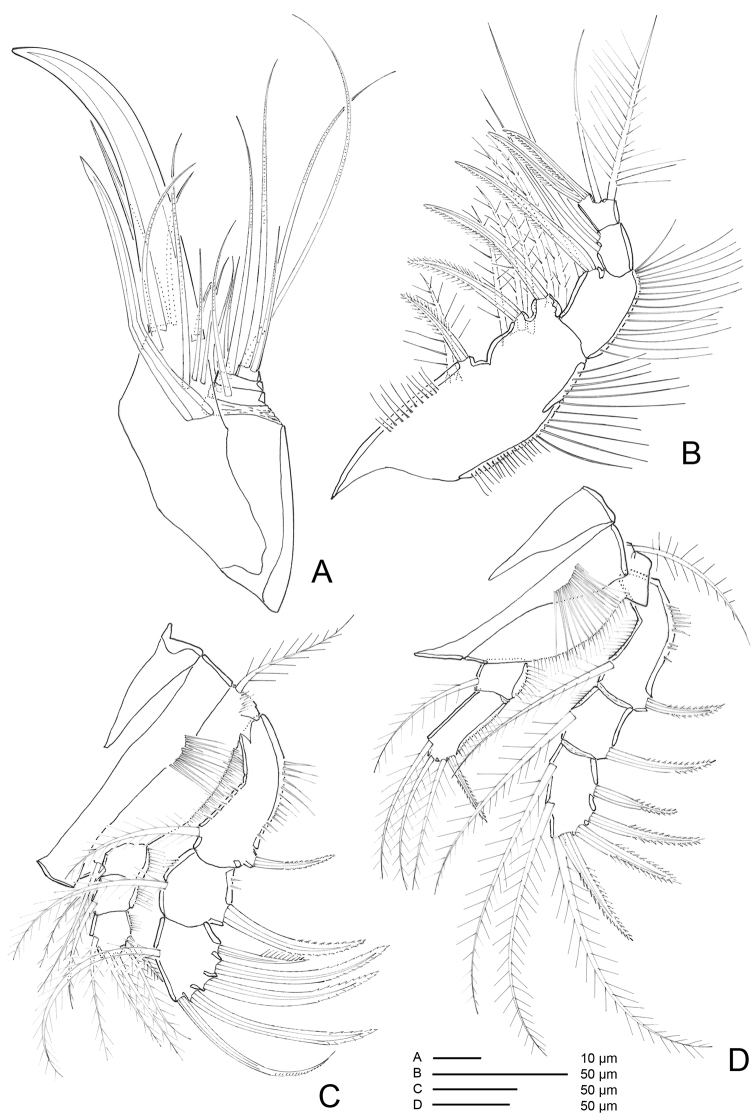
*Hase talpamorphicus* gen. et sp. n. Holotype (female) (M85/3, 1164): **A** Mx2 enp **B** Mxp **C**P1 **D**P2.

*Mx2* (Figs 15D, 16D, 17C, 18A) with syncoxa fused to allobasis and 5-segmented enp. Syncoxa with four endites; proximal coxal endite with five setae (one naked and four pinnate); distal coxal endite almost completely incorporated into syncoxa, with three pinnate setae; proximal basal endite with three setae (two naked and one pinnate); distal basal endite with two naked setae and a pinnate spine. Enp-1 endite forming strong claw; accessory armature consisting of two naked setae, one spine and one tube pore; armature of fused enp-2 represented by three naked setae and one spine. Free enp 3-segmented with armature formula: I-[claw; 3 and tube pore], II-[4]; III-[2], IV-[2], V-[4].

*Mxp* (Figs 17D, 18B) with elongated protopod and 2-segmented enp. Protopod with rows of spinules along inner and outer margins; syncoxa with three endites; proximal endite with one bipinnate seta and one bipinnate spine; second endite with three bipinnate setae and one bipinnate spine; distal endite with two bipinnate setae and one bipinnate spine; basal endite represented by one naked seta and one unipinnate spine. Enp with armature formula: I-[2], II-[two unipinnate spines + one naked seta + one bipinnate seta].

**Figure 19.**
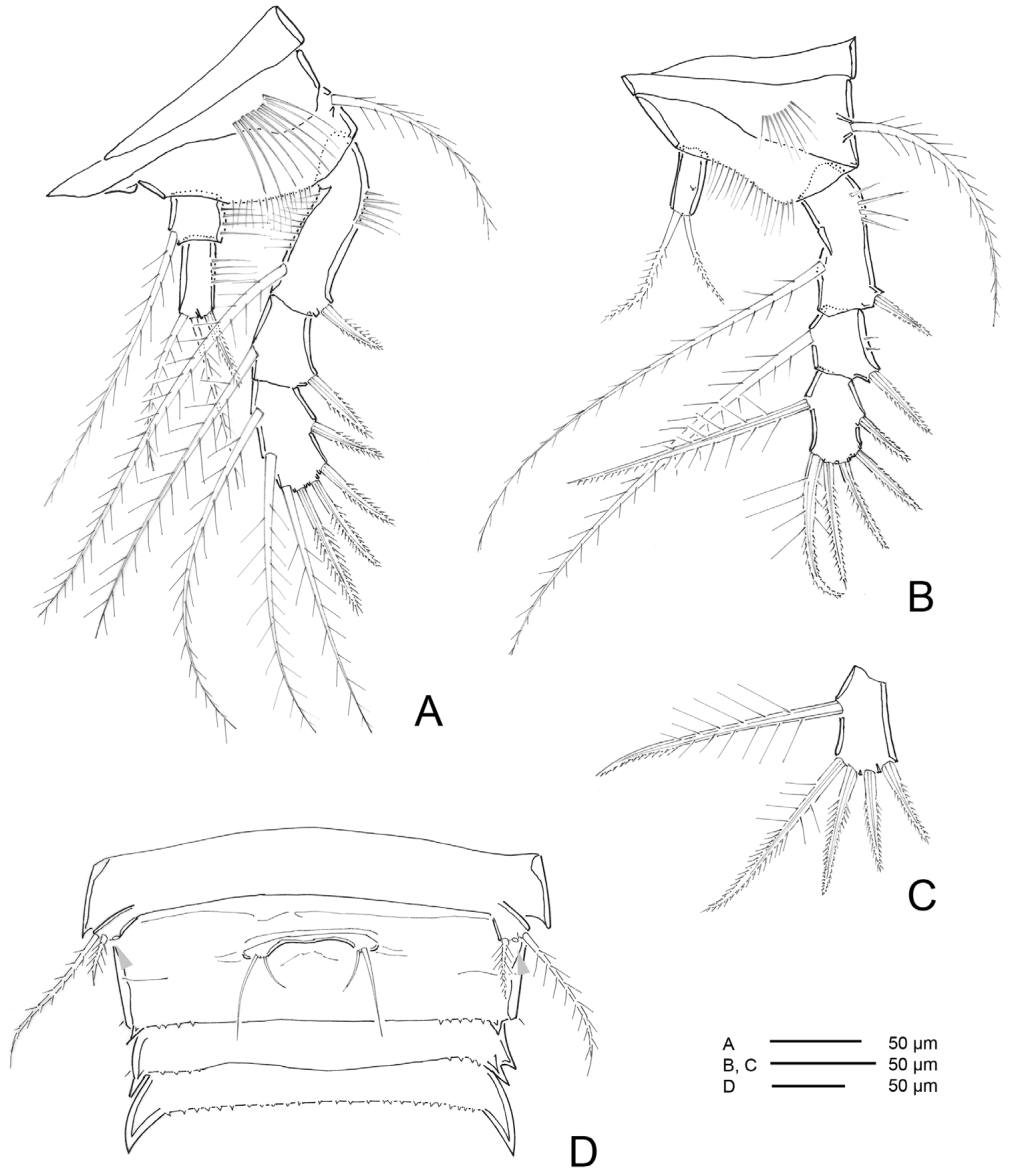
*Hase talpamorphicus* gen. et sp. n. Holotype (female) (M85/3, 1164): **A** P3 **B**P4 **C** variable P4 exp-3 found on the other side of the same specimen **D** P5, double genital somite and following urosomites.

**Figure 20.**
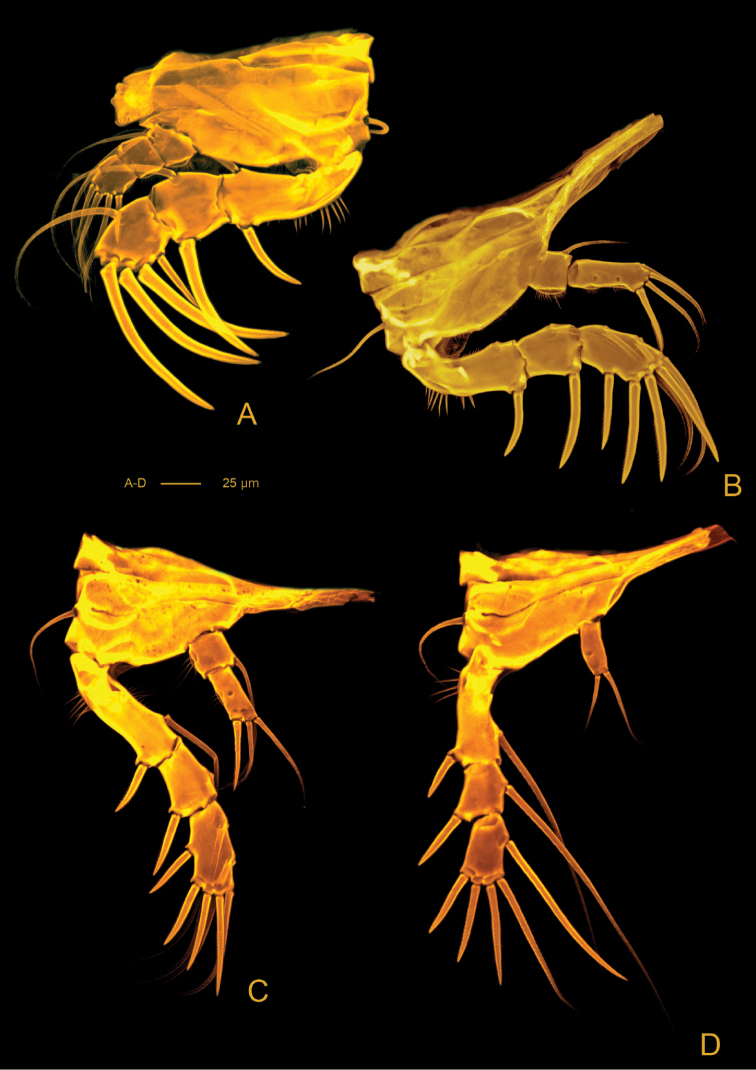
*Hase talpamorphicus* gen. et sp. n. Confocal laser scanning images. Holotype (female) (M85/3, 1164): **A** P1 **B** P2 **C** P3 **D** P4.

*Pereopods* (Figs 18C, D, 19A–C, 20A–D) biramous and flattened; exp and enp bent inwards, especially on P1 and P2. Praecoxa without ornamentation. Coxa with row of spinules along distal margin (P1) or anterior surface (P2-P4). Basis with (P1) or without (P2-P4) bipinnate seta on outer proximal corner, with bipinnate seta on inner distal corner, ornamentation consisting of patches of setules along outer (P1) and distal margins. Exp 3-segmented; exp-1 with rows of setules along inner and outer margins, exp-2 with rows of setules along inner (P1, P2) and outer margins (P1, P4). Enp 3-segmented (P1), 2-segmented (P2, P3) and 1-segmented (P4); enp- 1 with rows of setules along outer margin (P2, P3) or naked (P4). P1-P4 spine and setal formulae as follows:

**Table:** 

	Exp	Enp
P1	I, 1; I, 1; II, II+1, 1	0, 1; 0, 1; I, 2, 2
P2	I, 1; I, 1; II, II+1, 2	0, 1; I, 2, 1
P3	I, 1; I, 1; II, II+1, 2	0, 1; I, 2, 0
P4	I, 1; I, 1; II, II+1, I	0, 2, 0

*P5* (Figs 13E, 19D). Protopod fused to supporting somite, pointing outwards. Exp with three bipinnate setae.

Male unknown.

####### Remarks.

P4 exp-3 bears two outer spines in the normal condition (formula [II, II+1, I], two females). However, in one female, P4 exp-3 displayed [II, II+1, I] on one side and [I, II+1, I] on other side (Fig. 19C)

In the juvenile CV, segmentation and armature of P1-P4 as in *Hase lagomorphicus* gen. et sp. n.

## Discussion

### Taxonomic discussion and phylogenetic position within the Aegisthidae

According to Seifried and Schminke (2003), the monophyly of Aegisthidae is supported by the following female autapomorphies: 1) anal somite elongated, tapering posteriorly; 2) caudal rami more than twice as long as wide; 3) antennule of female 8-segmented; fusion of Oligoarthra segments 3 and 4; 4) antenna with allobasis or incomplete basis; 5) enp-2 laterally with one spine (III) and two setae (2 + 4), spine I lacking; 6) endopod of mandible of one large segment and at least two times longer than wide; 7) proximal segment of exopod elongated, considerably longer than remaining segments and at least 3 times longer than wide; 8) epipodite of maxillule represented by two setae; 9) exopod of maxillule reduced in size with three setae; endopodal element 11 of allobasis of maxilla developed as large, strong spine inserted on posterior surface; 10) P5 without endopodal lobe.

*Hase* gen. n. can be included within Aegisthidae on account of the above mentioned apomorphies 5, 6, and 10. Species of Aegisthinae are derived Cerviniinae and Cerviniopseinae (Seifried and Schminke 2003). Cerviniinae and Cervinopseinae are paraphyletic and as such, are defined by plesiomorphies. Therefore, *Hase* gen. n. cannot be placed within any of the subfamilies on the account of synapomorphies and its taxonomic position must be typological and on account of the close proximity to one of the taxa composing a given subfamily.

*Hase* gen. n. has an antenna with four-segmented exp and could be included within both Cerviniinae and Cerviniopseinae. However, Cerviniinae and Cerviniopseinae are to date separated according to the degree of divergence of the caudal rami (see Boxshall and Halsey 2004). Within Cerviniopseinae the caudal rami are closely appressed along the entire length. *Hase* gen. n. is included within the Cerviniinae due to the presence of a more or less divergent caudal rami. Within this subfamily, *Hase* gen. n. is the adelphotaxon of *Cerviniella.* In *Cerviniella* the three segmented exp P1-P4 is absent and the limbs are much transformed as an adaptation to a burrowing life within the sediment. They share a sturdy body (sy), and the exp-1 to 3 of P1-P3 are heavily built, transformed into digging limbs (sy), with strong outer and distal spines/setae (sy). When in resting position, the exopodite bends against the basis on at least the P1 and P2 (sy). They also share a 2-segmented enp on the P2 and P3 (sy), and a reduced P5 (sy).

In *Cerviniella* the whole exopod or the exopodite-2 and 3 are fused on the P1-P3 (sy), keeping the inner and outer armature of the original segments, the endopodite of P1 is never 3-segmented (sy) and the P4 undergoes a further reduction both in segmentation and/or armature of the exopod and endopod (sy) (viz. Kihara and Martínez Arbizu 2012). The strongest armature occurs on P2 (sy) and P3 (sy), with the P2 somite showing a very large proximal region of weakly sclerotized cuticle. Within *Hase* gen. n. the armature is more developed on the P1 (sy). They are longer and stronger on P1 and P2; shorter yet stout on P3 and P4. The P1, although keeping the plesiomorphic 3-segmented exopodite and endopodite, have the outer and distal elements transformed into strong and long spines (sy), kept as stiff setae on *Cerviniella* (pl). *Hase* gen. n. has one or two strong and long spines on the inner margin of the exopodite-3 of P4 (sy). The P5, which is fused to the somite (sy), is stalked and with three distal setae (sy). Additionally, the anal somite of *Hase* gen.n. is subquadratic, slightly tapering posteriorly, wider than longer (sy) and the caudal rami is one of the shortest yet described for this family (sy), with spiniform setae I to III (sy).

Interestingly, the same morphology of the P5, telson and furca is depicted by Brotskaya (1963) in the deep-sea genus *Paracerviniella*. This author briefly described *Paracerviniella* based on a male specimen only, as follows: Body without outgrowths. The first thoracic somite not completely separated from the cephalothorax. Postero-lateral corners of body somites, except for the first thoracic, drawn into pointed outgrowths. The posterior edge of all somites, except the anal, armed with a number of small teeth. Furcal rami 1.5 times shorter than the anal somite, width at the base one and a half times less than the length. The first antenna six-segmented, with two enlarged basal segments; the second, third and sixth segments of the male with sausage-like sensory cylinders, the fourth segment with a hooked spine, the fifth segment with two sensory cylinders of the usual structure. Both branches of P1-P4 triple-segmented. Endopodite of P1 and P2 with clawed spine at the distal part. P5 and P6 1-segmented with three apical bristles.

Most of these characters are not informative enough to allow the inclusion of *Paracerviniella* within any monophyletic clade within the Aegisthidae. With exception of the clawed spine present on the endopodites of P1 and P2, the P5 morphology and armature and maybe body ornamentation, the remaining characters are gender-linked or plesiomorphic within the family. In addition, the illustration of some characters that could be informative, such as the mouthparts, is insufficient. Considering this and on the absence of females, we cannot address in what degree *Hase* gen. n. and *Cerviniella* are phylogenetically related to *Paracerviniella.*

The main differences in morphology of *H. lagomorphicus* gen. et sp. n. and *H. talpamorphicus* gen. et sp. n. are summarized on Table 2. The somite bearing P3 and P4 has latero-distal spiniform processes in *H. talpamorphicus* gen. et sp. n. and smooth in *H. lagomorphicus* gen. et sp. n.. The antenna is armed with three stout spines on the lateral inner margin in *H. talpamorphicus* gen. et sp. n. and two proximal setae in *H. lagomorphicus* gen. et sp. n.; the distal outer element is a spine in *H. talpamorphicus* gen. et sp. n. and a seta in *H. lagomorphicus* gen. et sp. n., the three outer endopodal elements fused at the basis are represented by three setae in *H. lagomorphicus* gen. et sp. n. and two setae and a short and blunt spine in *H. talpamorphicus*. P4 exp-3 has two long and strong spines on the inner margin in *H. lagomorphicus* gen. et sp. n. and one spine in *H. talpamorphicus* gen. et sp. n.

The shape of the gonopores and the position of the copulatory pore as they are depicted by the CLSM (Figs 3C, 13E) revealed to be important characters for the separation of the two species. The copulatory pore is completely visible in *H. talpamorphicus* gen. et sp. n., whereas it is covered by a proximal flap and pointing posteriorly in *H. lagomorphicus* gen. et sp. n. The depression in which the copulatory pore is inserted is less developed in *H. lagomorphicus* gen. et sp. n. than in *H. talpamorphicus* gen. et sp. n. Finally, the operculum covering the gonopores is medially depressed in *H. lagomorphicus* gen. et sp. n. and straight in *H. talpamorphicus* gen. et sp. n.

**Table 2.** Distinctive characters of *Hase lagomorphicus* gen. et sp. n. and *Hase talpamorphicus* gen. et sp. n.

		*Hase lagomorphicus* gen. et sp. n.	*Hase talpamorphicus* gen. et sp. n.
Lateral margins of 3^rd^ and 4^th^ pedigerous somites		Smooth (Fig. 2A–B).	Expanded posteriorly forming hook-like projections laterally (Fig. 12A–B).
Rostrum	Tip	Rounded; with tuft of spinules along distal margin and with pair of sensilla near apex. (Fig. 2A).	Slightly bifid; with tuft of spinules along distal margin, with pair of sensilla and midventral tube-pore near apex (Fig. 12A and C).
A1	Segment II	8 setae (Fig. 4A).	7 setae + 2 missing elements (Fig. 14A).
	Segment III	10 setae + (1 seta+ ae) (Fig. 4A).	12 setae + (1 seta + ae)] (Fig. 14A).
A2	Enp medial armature	4 setae and 1 spine (Fig. 4B).	1 seta and 3 spines (Fig. 14B).
	Enp apical armature	3 spines, 1 seta and 3 elements fused basally (2 long setae medially unipinnate, and 1 smooth seta) (Fig. 4B).	4 spines, 1 seta and 3 elements fused basally (1 bipinnate seta, 1 unipinnate seta and 1 small flattened seta) (Fig. 14B).
	Exp-4	2 setae (Fig. 4B).	3 setae (Fig. 14B).
Md	Enp	3 lateral and 6 apical setae (Fig. 6A (a3)).	3 lateral and 7 apical setae (Fig. 16A).
Mx1	Arthrite	2 setae on anterior surface, 7 spines along distal margin, 4 setae on the aboral margin (Fig. 6C).	2 setae on anterior surface, 7 spines and 3 setae along distal margin, 2 setae on posterior surface. (Fig. 16B, C).
	Coxa endite distal armature	5 setae (Fig. 6C(c1 and c2)).	6 setae (Fig. 16B).
	Enp incorporated to basis	2 setae (Fig. 6C(c3)).	3 setae (Fig. 16B).
	Exp	3 setae (Fig. 6C(c4)).	2 setae (Fig. 16B).
Mx2	Enp-1 endite	2 setae, 1 spine and 1 claw-like spine (Fig. 6B(b5)).	2 setae, 1 spine and 1 tube pore (Fig. 18A).
	Enp-2	3 setae (Fig. 6B(b6)).	3 setae and 1 spine (Fig. 18A).
	Enp-5	3 setae (Fig. 6B(b7)).	4 setae (Fig. 18A).
Mxp	Syncoxal endites (proximal to distal)	1 seta and 1 spine, 2 setae and 1 spine, and 1 seta and 1 spine (Fig. 6D).	1 seta and 1 spine, 3 setae and 1 spine, and 2 setae and 1 spine (Fig. 17D).
	Enp-2	1 spine and 3 setae (Fig. 6D).	2 spines and 2 setae (Fig. 17D).
P4	Exp-3	II, II+1, II (Fig. 10B).	II, II+1, I (Fig. 19B).
	Enp	0, 2, I (Fig. 10B).	0, 2, 0 (Fig. 19B).
P5	Exp	1 seta, 1 spine and 1 missing element (Fig. 4E)	3 setae (Fig. 19D).
Genital Field	Copulatory pore	Slightly covered by a proximal flap, pointing posteriorly, located in a soft median depression (Fig. 3C).	Completely visible, not covered by a proximal flap as observed for the previous species, located in a well-developed median depression (Fig. 13E).
	Gonopores	Covered by medially depressed operculum (Fig. 3C).	Covered by a straight operculum (Fig. 13E).

### CLSM vs. SEM technology

There are some important differences among the scanning microscopy systems that produce high quality imaging, especially regarding to the subsequent fate of the specimens and the resolution limits. Some image systems (e.g., SEM) inevitably destroy type specimens; CLSM is highly desirable in this aspect as the studied specimen remains intact. According to Kamanli et al. (2017), the images obtained by CLSM are comparable in quality to SEM at the same magnifications, and the technique offers a 3D data set. In addition, the sample preparation routine for CLSM is simpler than that for SEM, it is practically a non-destructive method, and allows the study of hydrated material. It is difficult to establish a good SEM protocol for the study of miniaturized body parts of small macrofauna and meiofaunal specimens. Not infrequently they can be lost during manipulation, damaged before any observations are made (Michels 2007), or rendered unusable and in vain even during later processes such as coating in which the structure can become over-coated. CLSM also allows the appendages to be manipulated within the mounting medium to offer views of the specimen from multiple angles, which can be problematical to achieve using SEM since some viewpoints may be inaccessible due to the way that the specimen is mounted and the tilt limitations in SEM (Kamanli et al. 2017). After scanning, the material can be recovered intact and kept as a voucher. An example where CLSM is advantageous in the present species description is the dorsal (5B) and outer (5C) view of the same A1. In addition it offers a clear view of the natural 3-dimentional state of the antenna and the exact position of overlapping A1 setae, an arduous task during the traditional drawings of this structure. The continuous technological advancements in the field of microscopy are reducing the resolution gap among the different technologies. The resolution of SEM is approximately 10 nm whereas confocal microscopes have the potential to resolve microstructures in the 50 to 100 nm range (Schrader and Hell 1996). Practically, CLSM has reached a resolution comparable to SEM (Butler et al. 2010). In many situations, enhancing resolution beyond this range does not result in an increase in useful biological information about the specimen (St. Croix et al. 2005). Now, even for the smallest meiofaunal larvae, this level of resolution is more than sufficient to fully capture and catalogue the most minute external details such as pore morphology or individual setal ornamentation.

### The importance of digital image acquisition in taxonomy

Recent papers have highlighted the importance of image acquisition in taxonomy (e.g., Michels 2007, Neusser et al. 2009, Neusser et al. 2011, Faulwetter et al. 2013, Akkari et al. 2015). Garraffoni and Freitas (2017) argued that the International Code of Zoological Nomenclature should be modified to allow, in some cases, as in the study of rare or soft-bodied meiofaunal organisms, the deposit of high quality photographs and videos as Type material. This proposal has met with strong opposition among some researchers (Dubois 2017, Rogers et al. 2017). The evolution of optical systems has led to the exponential increase in the use of high quality imaging systems in all fields of biology, including taxonomy. Our opinion in this debate is that the image quality obtained by scanning through either CLSM, SEM or Micro CT is so high, that we should consider how viable it is to designate a photomicrography as Type material. Although this may sound provocative, we must consider that a well-curated image lasts potentially forever, whereas the type specimen, especially when we take into account small macrofauna and meiofaunal groups, may deteriorate fast during study or even when mounted on “permanent” slides, those have a half-life of only few decades or centuries. Diminution of trained museum staff to maintain collections only exacerbates this problem and highlights the need to seek alternative solutions to record and study taxonomically the world’s biodiversity (Decker et al. 2018). Hence, the use of CLSM and other high quality image acquiring systems should be considered not only as complimentary evidence to a taxonomical study. In some cases, the images generated should be also considered if not the type alone, at least part of the type series.

## Conclusions

This contribution highlights the diversity of exquisite bauplans in deep-sea copepods and the broad distribution of a meiobenthic crustacean genus in the Atlantic Ocean basins. Additionally, it is a showcase on how confocal microscopy can assist in providing a better and more accurate description of small macrofaunal and meiofaunal organisms. We favour the inclusion of digital media at least as a component of the type series and we encourage the discussion for setting standards for such data. Additional studies and sampling effort must be continued to find the male of the genus *Hase* gen. n. to improve comparisons with *Paracerviniella* and *Cerviniella.*

## Supplementary Material

XML Treatment for Hase

XML Treatment for Hase lagomorphicus

XML Treatment for Hase talpamorphicus
